# Heterosynaptic Memtransistors Based on Switching Operation Mechanism Using Designed Organic/Inorganic Heterostructures for Neuromorphic Electronics

**DOI:** 10.1002/advs.202517149

**Published:** 2026-01-05

**Authors:** Taek Joon Kim, Hye Lim Jeong, Sang Wook Song, Dayeong Kwon, Sang‐hun Lee, Jinsoo Joo

**Affiliations:** ^1^ Department of Physics Korea University Seoul Republic of Korea

**Keywords:** heterosynapses, memristors, memtransistors, MoS_2_, neuromorphics, organic semiconductors

## Abstract

Memtransistors using low‐dimensional semiconductors represent a promising gate‐tunable heterosynaptic architecture for neuromorphic computing. However, active layers of these devices have not yet been artificially designed or controlled. In this study, gate‐pulse‐tunable heterosynaptic neuromodulation is achieved using memtransistors with organic semiconductor tris(4‐carbazoyl‐9‐ylphenyl)amine (TCTA)/MoS_2_ heterostructures designed via energy‐band engineering and bottom‐contact architecture. Memristive switching is realized through distinctive low‐ and high‐conduction states with a switching ratio of 10^2^, modulated by gate pulses. As the gate voltage (*V*_G_) decreases from +30 to −30 V, the memristive hysteresis for the bottom contact TCTA/MoS_2_ FET without post‐treatment and an h‐BN insulating layer appears at *V*_G_ = −15 V and broadens with an increasing switching ratio. Intriguingly, as *V*_G_ becomes increasingly negative (*V*_G_ < −15 V), trap‐related space‐charge‐limited conduction becomes dominant. Non‐volatile heterosynaptic behavior is mimicked by drain pulses and modulated by gate‐pulse polarities. Applying gate‐pulse only, analogous responses are observed in synaptic modulation with time constants of 100 ms for potentiation and 60 ms for depression. The design of multi‐functional memory and realization of gate‐pulse‐tunable memtransistors using nanoscale TCTA/MoS_2_ can promote energy‐efficient, tunable, and reliable heterosynaptic neuromorphic electronics.

## Graphical abstract

Heteroysynaptic memtransistors are fabricated using a combination of π‐conjugated organic semiconductor TCTA and TMDC MoS_2_ heterostructure with bottom‐contact architecture. Non‐volatile heterosynaptic and homosynaptic functions such as long‐term plasticity and spike‐timing‐dependent plasticity are emulated by drain and gate pulses based on conduction mechanism switching. Elaborately designed framework can offer reliability and reproducibility for artificial neuromorphic computing systems.

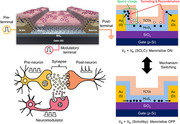

## 1 Introduction

Memtransistors based on a carefully selected semiconductor combination and three‐terminal architecture (source, drain, and gate electrodes) can enable energy‐efficient neuromorphic computing via heterosynaptic plastic neural networks [1, 2, 3, 4]. Vertical‐type memristors with top and bottom electrodes have been intensively studied for their dual functions as memory and resistors in non‐volatile resistive random‐access memory and neuromorphic computing applications [5, 6, 7, 8, 9, 10]. These devices can control resistance states by memorizing previous electrical inputs, thereby mimicking the synaptic functions in biological neural networks [11, 12, 13, 14, 15, 16].

Memory‐based neuromorphic devices represent a fundamental advancement toward hardware implementation capable of spiking‐neuron operations. Moving beyond their function as simple memory elements, these devices are uniquely designed to emulate biological synapses, facilitating both synaptic weight storage and neuron firing functionalities that effectively mimic the behavior of the human brain. These neuromorphic architectures offer significant advantages over conventional CMOS‐based neural networks, such as enhanced energy efficiency and the ability to realize highly integrated spiking neural networks (SNNs). Furthermore, their operational performance can be critically optimized through various in situ learning algorithms [15, 17, 18, 19]. Memory‐type neuromorphic devices also provide the foundation for advanced sensory‐perceptive interactive systems. They can directly convert external sensory stimuli into electrical spikes, thereby achieving a unified hardware platform that integrates sensing, signal processing, and decision‐making [15].

Early research on memristive switching focused on metal/transition metal oxide (TMO; e.g., TiO_x_, TaO_x_, AlO_x_, CuO_x_)/metal vertical structures, examining the atomic‐scale movement of metallic ions and vacancies [20, 21, 22, 23]. Accurate atomic‐level control of charge carriers in the metal/TMO/metal configuration enabled various synaptic functions [24, 25]. However, these TMO‐based frameworks should be improved for multi‐functionality tuning of the channel conductance via gate bias and/or pulse for advanced artificial synapses of neuromorphic electronics. To overcome these limitations, 2D transition metal dichalcogenides (TMDCs), such as MoS_2_, WS_2_, WSe_2_, and MoTe_2_, along with graphene, MXene, and h‐BN, have been explored for the development of atomically scaled memristors with vertical structures [26, 27, 28, 29]. In these 2D‐TMDC‐based memristors, featuring vertical and heterojunction (HJ) structures with top and bottom electrodes, memristor switching and synaptic functions were analyzed considering mechanisms such as vacancy migration [30, 31, 32], filament formation [33, 34], phase transition [35, 36], and Schottky to direct tunneling charge transport [37]. Owing to their scalable atomic thickness, these memristors exhibited outstanding energy efficiency with low turn‐on voltages [38, 39]. However, the precise control of charge carriers, ions, defects, trap states, and vacancies remains crucial for achieving the desired performance in neuromorphic electronics. Moreover, the simple two‐terminal structures of memristors using 2D TMDCs as active layers cannot effectively achieve hetero‐integration and multi‐functionality, which are essential for future neuromorphic synaptic elements.

Memtransistors with lateral structures, such as field‐effect transistor (FET) configurations, have been fabricated using monolayer MoS_2_ grown by chemical vapor deposition (CVD) [1, 40]. In such devices, memristor characteristics have been associated with defect migration at grain boundaries in MoS_2_. Although these memtransistors benefited from atomic thickness and multi‐tunable electric signals, precise control of defects and irregular grain boundaries associated with the polycrystalline nature of CVD‐grown monolayer MoS_2_ remained unresolved. Huh et al. reported gate‐pulse‐tunable artificial heterosynaptic architectures using MoS_2_‐channel memtransistors with oxidation‐induced defects [3]. Memristive switching between source and drain was achieved by defect‐mediated space‐charge‐limited conduction (SCLC), modulated by gate voltages. Synaptic weights in three‐terminal MoS_2_ memtransistors were programmed with sub‐femtojoule drain and gate pulses. However, the memristor characteristics, including synaptic functions, strongly depended on the generation of oxidation defects on thin surfaces of MoS_2_ via UV‐ozone treatment. This necessitated precise and reproducible control of defect/trap states and spike‐timing‐dependent plasticity (STDP) learning.

Despite the considerable advances in memristors in recent years, improvements in power consumption, tunability, multi‐functionality, and reliability are required for future neuromorphic electronics. To implement artificial heterosynaptic multi‐functionality, the active layers of memtransistors must be sensibly designed using distinct low‐dimensional heterostructures (HSs) based on energy‐band engineering. Accurate modulation between the high‐resistance state (HRS) and low‐resistance state (LRS), plasticity, and depression can be realized through gate pulses in FETs using such HS‐based active layers. These abilities can enable artificial and precise control of charge currents corresponding to neuron signals. Specifically, heterosynaptic neuromodulation and neurotransmission between pre‐neurons and post‐neurons can facilitate the realization of neuromorphic electronics.

In this study, we fabricated three‐terminal memtransistors with a vertically stacked HS as an active layer consisting of an organic semiconductor tris(4‐carbazoyl‐9‐ylphenyl)amine (TCTA) donor and MoS_2_ acceptor. The TCTA/MoS_2_ HS demonstrated type‐II energy‐band alignment (EBA) and induced low and high current channels for the HRS and LRS. The Fermi‐energy level (*E*_F_) in the active layer of the FET could be tuned by gate bias, resulting in multi‐functionality for neuromorphic electronics. By combining a few‐layer MoS_2_ with an ordered nanometer‐thick TCTA layer, artificial synaptic devices with reproducibility could be developed. The bottom‐contact (BC) FETs incorporating the TCTA/MoS_2_ HS exhibited the distinct current channels via gate modulation, realizing the memtransistor with multi‐level configurations. Notably, non‐volatile synaptic behavior was successfully mimicked by drain pulses and modulated by the polarity of the gate pulses. Analogous responses were observed even without drain pulses (i.e., gate‐pulse only condition). To the best of our knowledge, such material selection, gate‐switchable conduction mechanisms, and multi‐input‐based operations within a single FET device using a *p*‐type‐organic/*n*‐type‐inorganic TCTA/MoS_2_ HS have not been reported in the literature. The proposed framework provides a promising platform for the design of artificial multi‐functional systems for neuromorphic computing.

## 2 Results and Discussion

### 2.1 Energy Band and Device Structures

**FIGURE 1:**
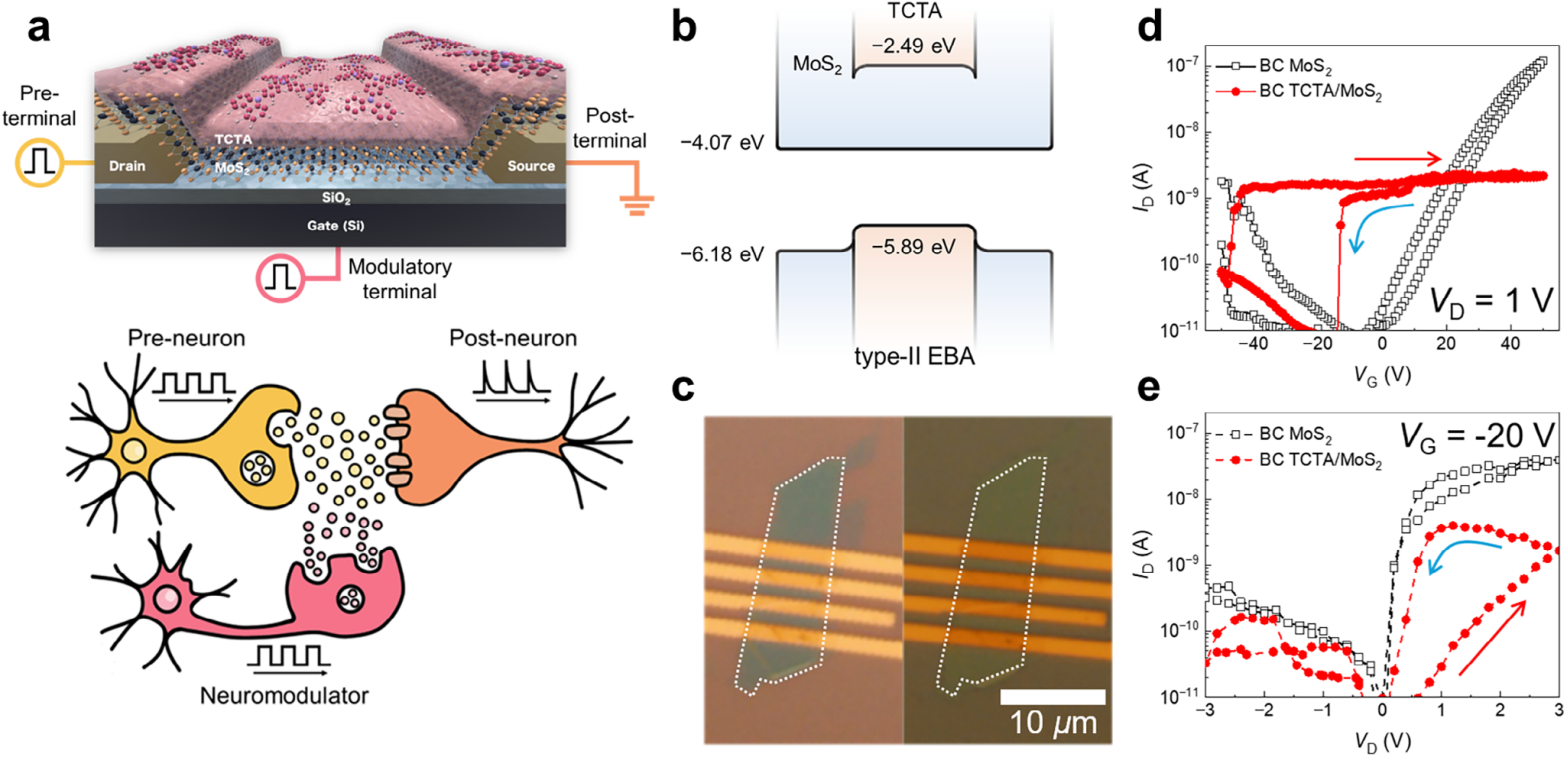
Bottom‐contact (BC) TCTA/MoS_2_ memtransistors. (a) Schematic of the BC TCTA/MoS_2_ memtransistor (top) and corresponding biological neural system (bottom). (b) Energy band alignment of the BC TCTA/MoS_2_ memtransistor. The TCTA energy band is shown inside the MoS_2_ layer to reflect the bottom‐contact structure of the TCTA/MoS_2_ memtransistor. (c) Optical microscopy image of the TCTA/MoS_2_ memtransistor. The white dotted lines represent sample boundaries. (d) *I*_D_–*V*_G_ transfer characteristic curves of the FETs, measured at *V*_D_ = 1 V for pristine MoS_2_ (open black markers) and TCTA/MoS_2_ (solid red markers). (e) *I*_D_–*V*_D_ output characteristic curves of the pristine MoS_2_ (open black markers) and TCTA/MoS_2_ (solid red markers) FETs measured at *V*_G_ = −20 V.

Figure 1a schematically illustrates the memtransistor (top) and corresponding heterosynaptic neural system (bottom). Based on the concept of the memtransistor, the drain electrode in the FET device corresponds to the pre‐neuron, the source electrode to the post‐neuron, and the gate electrode to the modulatory neuron. Pre‐ and modulatory impulses were simulated by applying voltage pulses to the drain and gate electrodes, respectively. The active layer of the memtransistor was composed of an HS formed by the organic π‐conjugated donor TCTA and inorganic 2D acceptor MoS_2_. As shown in Figure 1b, the TCTA/MoS_2_ HS exhibited a type‐II EBA. The highest occupied molecular orbital and lowest unoccupied molecular orbital levels of TCTA were estimated via ultraviolet photoelectron spectroscopy (UPS) and absorption spectroscopy (Figure S1). The valence band maximum (VBM) and conduction band minimum (CBM) of MoS_2_ were referenced from the literature [41]. Owing to the energy offset between the TCTA and MoS_2_ layers, electrons and holes could be easily transferred through the heterointerface. For the characteristics of the interface and of materials, X‐ray photoelectron spectra (XPS) data of pristine MoS_2_ and TCTA/MoS_2_ heterostructure, absorption and PL spectra of TCTA, AFM images with thickness analysis of MoS_2_ are shown in Figures S2–S4 and Table S1.

Figure 1c shows an optical microscopy image of the device. The channel length and width of the BC TCTA/MoS_2_ FET were 1.32 and 7.51 µm, respectively. Figure 1d shows the transfer characteristic curves (*I*_D_–*V*_G_) of the BC MoS_2_ FETs at *V*_D_ = 1 V before (open black markers) and after (solid red markers) hybridization with TCTA. The pristine BC MoS_2_ FET exhibited a high n‐type current with a threshold voltage (*V*_th_) near 0 V. In contrast, for the BC TCTA/MoS_2_ FET, *V*_th_ shifted to −50 V in the forward sweep (red arrow) and −15 V in the backward sweep (blue arrow), accompanied by a wide current saturation region and broad and hard hysteresis within the sweep cycle, as shown in Figure 1d. To comprehensively assess the charge transport mechanism, output characteristic curves (*I*_D_–*V*_D_) of the pristine BC MoS_2_ (open black markers) and BC TCTA/MoS_2_ (solid red markers) FETs were measured at *V*_G_ = −20 V (within the hysteresis range of −50 to −15 V) using a cyclic forward and reverse *V*_D_ sweep from 0 to 3 V (Figure 1e). The BC TCTA/MoS_2_ FET exhibited a distinct hysteresis loop with clear resistance switching, while the pristine BC MoS_2_ FET showed typical n‐type diode‐like behavior. Notably, such large hysteresis was not observed in the top‐contact (TC) FETs (Figure S5). In the BC FETs, the source–drain (S–D) electrodes contacted only the MoS_2_ layer, whereas in the TC FETs, the S‐D electrodes contacted both MoS_2_ and TCTA (Figures S5 and S6). Using a higher *V*_D_ = 2 V, transfer characteristic curves (*I*_D_–*V*_G_) similar to those in Figure 1d were observed (Figure S7a). The hysteretic output characteristic curves (*I*_D_–*V*_D_) were observed for the BC TCTA/MoS_2_ FET with negative gate voltages (−20 and −30 V), as shown in Figure S7b–h. The observed memristive switching behavior and gate‐tunable three‐terminal architecture of the BC TCTA/MoS_2_ FET could facilitate multi‐level configurations for heterosynaptic systems such as memtransistors.

### 2.2 Memtransistor Characteristics

**FIGURE 2:**
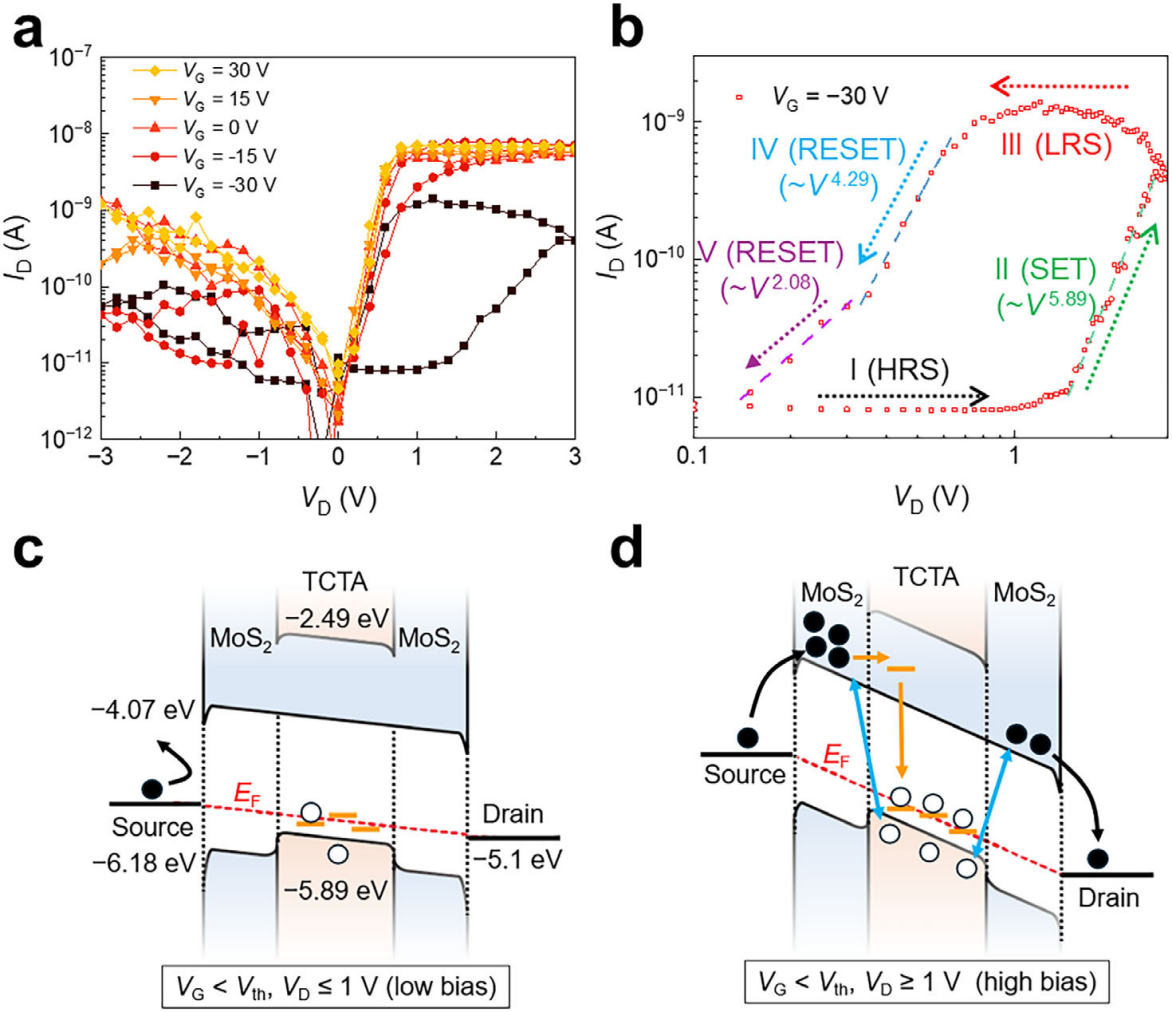
Gate tunability and charge transport mechanism of memristive switching characteristics. (a) *I*_D_–*V*_D_ output characteristic curves of BC TCTA/MoS_2_ memtransistors, measured at *V*_G_ = −30, −15, 0, 15, and 30 V. (b) Dual‐logarithmic plot of the *I*_D_–*V*_D_ curve at *V*_G_ = −30 V. Schematic energy‐band diagrams showing charge transport at *V*_G_ < *V*_th_ with (c) low and (d) high source‐drain bias (*V*_D_). Black, orange, and blue arrows represent charge transport, Shockley–Read–Hall, and Langevin recombination, respectively.

To explore gate tunability, the output characteristic curves (*I*_D_–*V*_D_) of the BC TCTA/MoS_2_ memtransistor were measured at various *V*_G_ (−30, −20, −15, −10, 0, 10, 15, 20, and 30 V), as shown in Figure 2a and Figure S7. As *V*_G_ negatively increased from +30 to −30 V, memristive hysteresis broadened with increasing the switching ratio up to 10^2^. It should be noted that no post‐treatment was performed to induce extrinsic traps in the MoS_2_ and TCTA layers, and no h‐BN insulating layer was adopted. These imply that the switching characteristics of our memtransistor are attributed to the hybridization with organic TCTA in the BC FET structure. Memristive switching was clearly observed in the BC TCTA/MoS_2_ FETs after the formation of the HS (Figure 2a). The memristive hysteresis characteristics were only observed for the BC TCTA/MoS_2_ FETs at high negative *V*_G_, not for the TC TCTA/MoS_2_ FETs (Figure S8).

The output characteristics of the BC TCTA/MoS_2_ memtransistor were analyzed through the power‐law relation (*I*_D_ ∝ *V*_D_^m^) based on the SCLC model [3, 42], described by the Mott–Gurney law [43, 44]:

(1) $$J_{\mathrm{SCLC}}=\frac{9}{8}\;\theta \mu \varepsilon_{0}\varepsilon_{r}\frac{V^{2}}{L^{3}},$$

where *J*, *θ*, *µ*, *ε*_0_, *ε*_r_, *V*, and *L* represent the current density, ratio of free to total carrier concentration, mobility, vacuum permittivity, relative dielectric constant, applied voltage, and channel length, respectively. Assuming the occupation of exponentially distributed traps, *J* can be described using the trap‐filled‐limited (TFL) SCLC model, described by the Mark–Helfrich law [45]:

(2) $$J_{\mathrm{TFL}}=e^{1-l}\;\mu N_{\mathrm{eff}}{\left(\frac{\varepsilon_{0}\varepsilon_{r}}{N_{t}}\frac{l}{l+1}\right)}^{l}{\left(\frac{2l+1}{l+1}\right)}^{l+1}\frac{V^{l+1}}{L^{2l+1}},$$

where *e*, *N*_eff_, and *N*_t_ denote the elementary charge, effective density of states, and total trap density, respectively. The parameter *l*, which represents the ratio of trap‐related characteristic energy to the measured thermal energy (i.e., *l* = *k*_B_ *T*_c_/*k*_B_ *T*), is typically larger than or equal to 1.

Figure 2b shows the dual‐logarithmic plot of *I*_D_–*V*_D_ in the range of 0.1–3 V at *V*_G_ = −30 V. Owing to the application of this negative *V*_G_, the Fermi level (*E*_F_) shifted downward toward the VBM (Figure 2c). Additionally, when *V*_G_ was lower than *V*_th_ = −15 V, electrons in MoS_2_ and holes in TCTA accumulated near the HJ interface (Figure S6a). Electrons (holes) could be transferred across the TCTA and MoS_2_ layers at the HJ through the Shockley–Read–Hall (SRH) trap‐intermediated tunneling process and Langevin recombination [46, 47]. In the case of *V*_D_ ≤ 1 V [Region I (HRS)], *I*_D_ remained below 10^−11^ A owing to the low charge density. As *V*_D_ increased further [Region II (SET)], additional charges were injected into the MoS_2_, leading to high charge concentration near the HJ interface (Figure 2d). These accumulated charges led to space‐charge formation, inducing TFL‐dominated charge transport with a slope of *m* = 5.89 in the plot of *I*_D_ ∝ *V*_D_^m^. In addition, these charges enhanced the interface recombination rate, resulting in the formation of a p‐type channel in TCTA. Therefore, in the *V*_G_ < *V*_th_ condition, owing to the formation of the TCTA channel (SET process), the charges near *E*_F_ were effectively transported through the n–p–n junction (MoS_2_–TCTA–MoS_2_), contributing to memristive behavior. During backward sweep (from *V*_D_ = 3 to 0 V), a highly saturated current was observed [0.7 V ≤ *V*_D_ ≤ 3 V, Region III (LRS)]. Subsequently, *I*_D_ rapidly decreased with a slope of *m* = 4.29 in *I*_D_ ∝ *V*_D_^m^ [0.3 V ≤ *V*_D_ ≤ 0.7 V, Region IV (RESET)], indicating reduced charge injection and tunneling rate. When *V*_D_ ≤ 0.3 V [region V (RESET)], trap‐limited SCLC behavior (*m* = 2.08) was observed owing to the presence of deep‐trapped charges in the TCTA and at the interface channel [3].

To further investigate the gate‐dependent charge transport through the interface, the trap density (*N*_t_) was estimated using gate‐dependent output characteristics in Figure S9. As *V*_G_ varied from −15 to −30 V, *V*_TFL_ (voltage with filled traps) and *N*_t_ increased from 0.36 V and 8.95 × 10^13^ cm^−3^ to 1.52 V and 4.14 × 10^14^ cm^−3^, respectively (Table S2). These results indicate that the negative gate bias enhanced both the current level and *N*_t_ in the active layer of TCTA/MoS_2_, suggesting a gate‐dependent switching mechanism.

**FIGURE 3:**
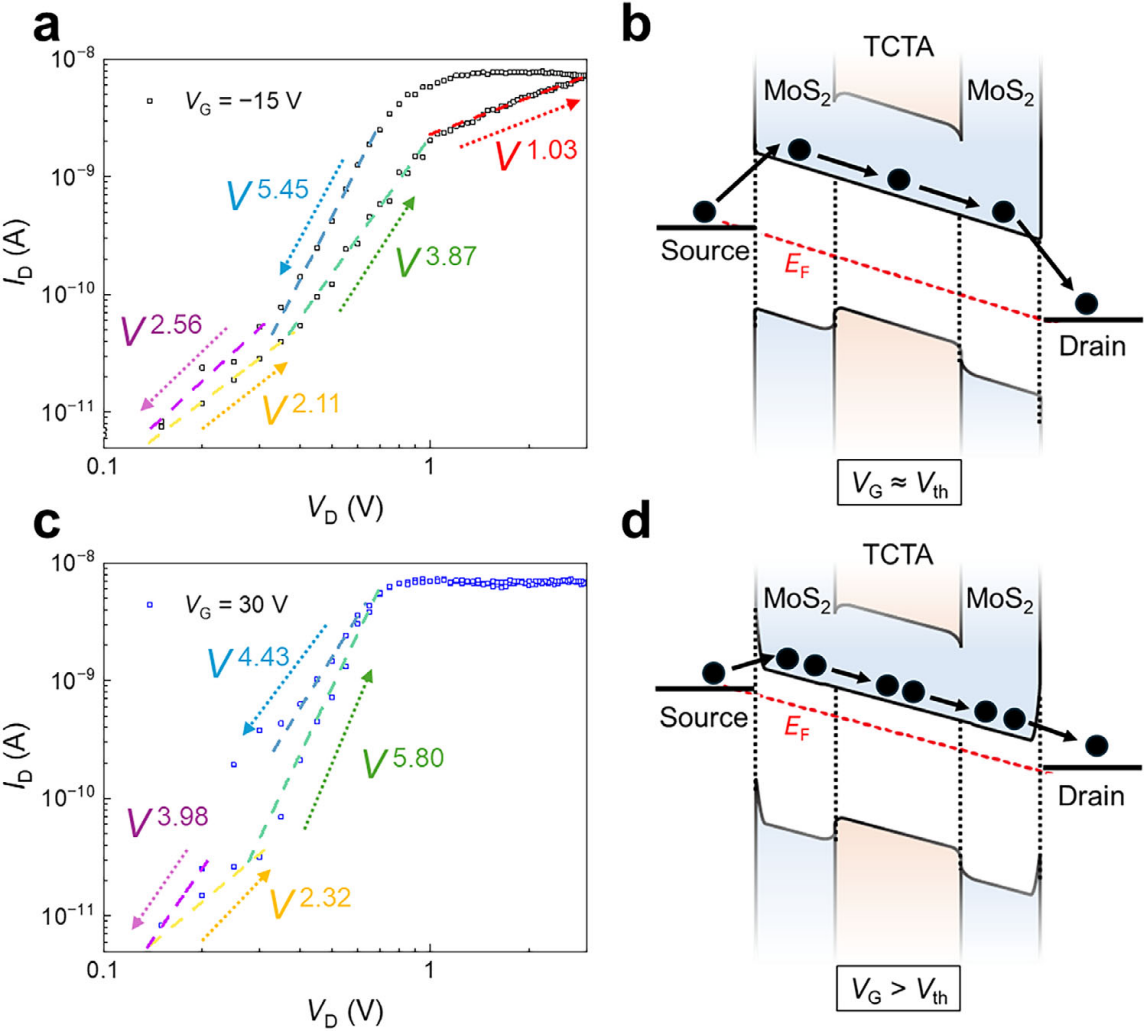
Gate‐tunable charge transport mechanism. (a) *I*_D_–*V*_D_ output characteristic curves of TCTA/MoS_2_ memtransistors at *V*_G_ = −15 V and (b) corresponding schematic of charge transport at *V*_G_ ≈ *V*_th_. (c) *I*_D_–*V*_D_ output characteristic curves of TCTA/MoS_2_ memtransistors at *V*_G_ = 30 V and (d) corresponding schematic of charge transport at *V*_G_ > *V*_th_.

The charge transport mechanism of the BC TCTA/MoS_2_ memtransistors was investigated through *I*_D_–*V*_D_ output characteristic curves and energy‐band models at *V*_G_ = −15 and 30 V, as shown in Figure 3. At *V*_G_ = −15 V, near the *V*_th_ (Figure 3a), *E*_F_ shifted slightly upward (Figure 3b). Charge transport was initially governed by SCLC (*m* = 2.11), followed by modest TFL conduction (*m* = 3.87), up to *V*_D_ = 1.0 V. At *V*_D_ > 1.0 V, *I*_D_ exhibited ohmic behavior (*m* = 1.03), suggestive of the saturation regime in SCLC [48]. Notably, under a low negative *V*_G_ (= −15 V), the hysteresis of the output *I*_D_ narrowed (Figure 3a). At *V*_G_ = 30 V (> *V*_th_; Figure 3c), *E*_F_ shifted upward toward the CBM of MoS_2_ (Figure 3d). In this regime, electrons were tunneled through the metal–semiconductor contact barrier, inducing n‐channel current in the MoS_2_ (Figure S6b). At *V*_G_ = 30 V, Schottky conduction dominated the charge transport mechanism. As *V*_G_ became increasingly negative, SCLC emerged as the dominant conduction mechanism, supporting the gate‐dependent charge transport model (Figure S10 and Table S3). At positive *V*_G_, *I*_D_ rapidly increased as *V*_D_ increased up to 0.7 V owing to the lowering (and/or narrowing) of the Schottky barrier. For *V*_D_ ≥ 0.7 V, *I*_D_ became saturated (*I*_sat_). Specifically, owing to the high *V*_D_, the S–D electric field induced the pinch‐off state in the MoS_2_ channel. The plot of *I*_sat_^1/2^ vs. *V*_G_ obtained from the *I*_D_–*V*_D_ curves at various positive *V*_G_ exhibited a linear relationship (Figure S11), consistent with the typical current saturation observed in FETs (*I*_sat_ ∼ *V*_G_^2^) [48]. Overall, the observed gate‐tunable memristive switching characteristics in BC TCTA/MoS_2_ memtransistors could be explained using the trap‐related SCLC model.

### 2.3 Control Devices and Neural Functions of Memtransistors

Analogous to long‐term plasticity (LTP) and STDP in biological synapses, the conductance of FETs can be modulated by consecutive voltage pulses applied to the drain terminal, a phenomenon known as homosynaptic plasticity [49]. Owing to the memristive switching characteristics of our three‐terminal TCTA/MoS_2_ memtransistors, heterosynaptic plasticity could be realized, with gate modulation influencing the underlying charge transport mechanism even without direct stimulation at the drain. The BC TCTA/MoS_2_ memtransistors studied herein mimicked the synaptic functions via tunable and non‐volatile changes in post‐synaptic current (PSC) in response to electrical stimuli. In this model, the drain, source, and gate terminals served as the pre‐neuron, post‐neuron, and modulatory neuron in biological neuron systems, respectively.

**FIGURE 4:**
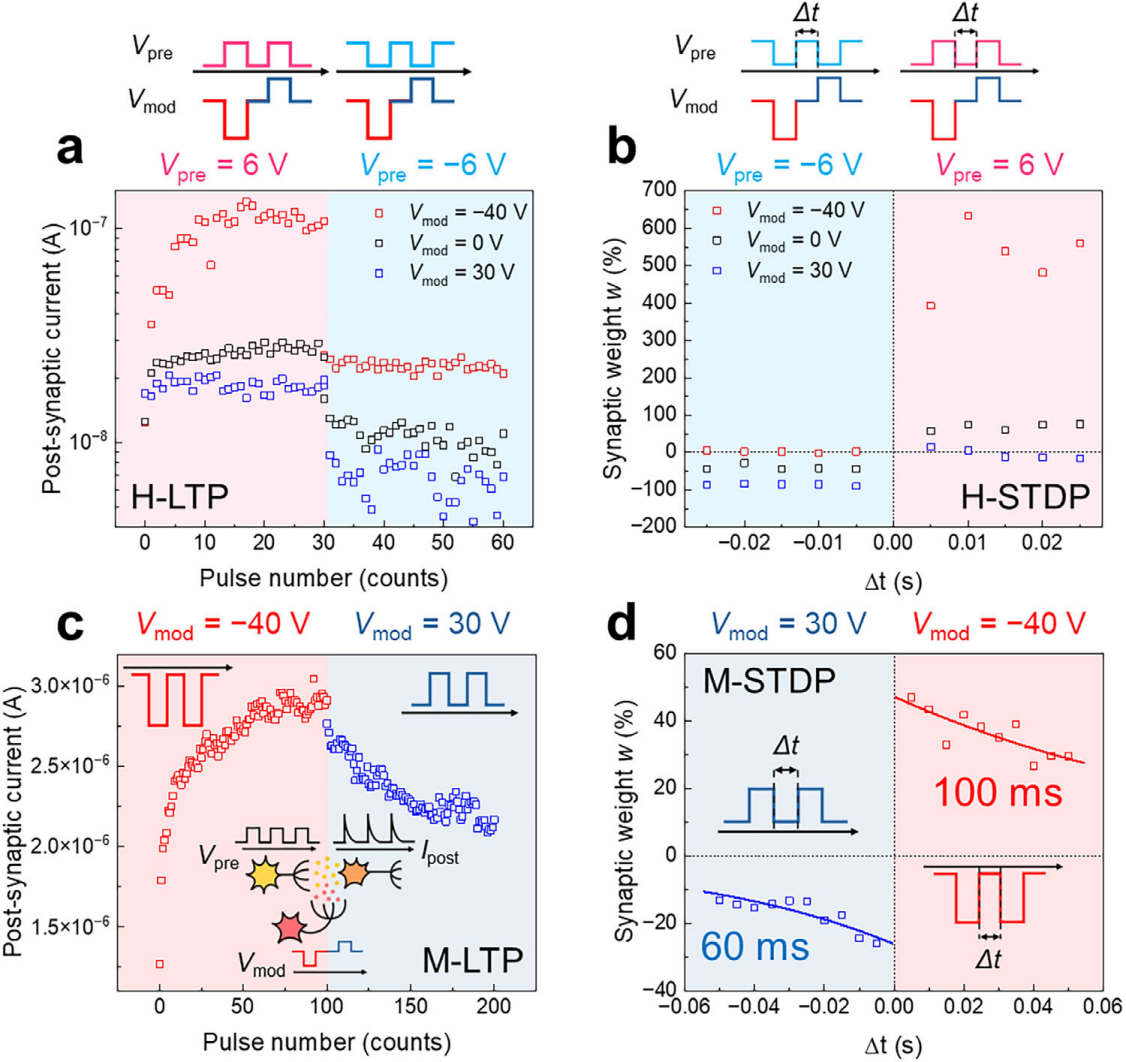
Heterosynaptic and modulatory‐induced homosynaptic characteristics of the TCTA/MoS_2_ memtransistors. (a) Heterosynaptic long‐term plasticity (H‐LTP) as a function of pre‐impulse (*V*_pre_) number with various modulatory impulses (*V*_mod_): −40 V (open red markers), 0 V (open black markers), and 30 V (open blue markers). (b) Heterosynaptic spike‐timing‐dependent plasticity (H‐STDP) as a function of the time interval (Δ*t*) between paired *V*_pre_ under different *V*_mod_: −40 V (open red markers), 0 V (open black markers), and 30 V (open blue markers). (c) Modulatory‐induced homosynaptic LTP (M‐LTP) as a function of pulse number under *V*_mod_ (over 100 pulses). (d) Modulatory‐induced homosynaptic STDP (M‐STDP) as a function of Δ*t* between paired *V*_mod_. H‐LTP and H‐STDP were measured using synchronous *V*_pre_ and *V*_mod_, whereas M‐LTP and M‐STDP were measured without *V*_pre_.

Figure 4 shows the heterosynaptic (H‐) and modulatory‐induced homosynaptic (M‐) characteristics along with their measurement conditions (Figure S12). During all measurements, the pulse width (*t*_width_) was fixed at 5 ms, and the read voltage pulse (*V*_read_) was set as −3 V. As shown in Figure 4a, the H‐LTP process was activated by applying 30 potentiation pulses to the drain (*V*_pre_ = 6 V), followed by 30 depression pulses (*V*_pre_ = −6 V). Without gate‐modulation pulses (*V*_mod_ = 0 V), the PSC increased only slightly during potentiation, whereas it decreased under depression (open black markers in Figure 4a). Interestingly, the PSC depended strongly on the polarity of *V*_mod_: the synaptic response was enhanced at *V*_mod_ = −40 V (open red markers in Figure 4a). In contrast, at *V*_mod_ = 30 V, the PSC was suppressed (open blue markers in Figure 4a). These polarity‐dependent characteristics are qualitatively consistent with the gate‐dependent output characteristics for the BC TCTA/MoS_2_ memtransistors (Figure 3). Furthermore, the H‐STDP process was investigated using the same values of voltage and pulse widths as those in the LTP process, albeit with a different pulse sequence (Figure 4b). In memristive systems, the synaptic weight *w* represents the synaptic strength, that is, the strength of connection between pre‐ and post‐neurons in biological systems, and it can be expressed as: [50, 51]

(3) $$w=\left(I_{\mathrm{STPD}}-I_{0}\right)/I_{0}\;$$

where *I*_0_ and *I*_STDP_ denote the measured current before and after applying paired pulses, respectively, imitating the biological activations of pre‐ and post‐neurons [1]. In neural systems, when the post‐neuron is activated after the pre‐neuron, i.e., the time interval (Δ*t* = *t*_post_−*t*_pre_) of the impulses is positive (Δ*t* > 0), the connection of the two neurons is strengthened. For Δ*t* < 0, the two neurons are not connected, resulting in the weakening of synaptic connections. These processes are key mechanisms in neural systems for energy‐efficient learning and memorizing. In addition, *w* as a function of Δ*t* is related to the activity‐dependent synaptic behavior, described as an exponential decay function [51, 52],

(4) $$w_{+}=A_{+}+\exp\left(\left(-t\right)/\tau_{+}\right)\;\mathrm{and}\;\;w_{-}=A_{-}+\exp\left(\left(-t\right)/\tau_{-}\;\right)$$

where *A* and *τ* denote the maximum change and time constant, respectively, and subscripts + and − correspond to positive and negative Δ*t*, respectively. Notably, at *V*_mod_ = −40 V (red markers in Figure 4b), *w* exhibited enhanced responses during potentiation (Δ*t* > 0) and weak responses during depression (Δ*t* < 0), compared with those in the case with *V*_mod_ = 0 V (black markers in Figure 4b). In contrast, at *V*_mod_ = 30 V (blue markers in Figure 4b), *w* reduced in the potentiation region (Δ*t* > 0) but was enhanced in the depression regime (Δ*t* < 0). These results clearly demonstrate polarity‐dependent synaptic modulation, in accordance with H‐LTP, suggesting the ability of the BC TCTA/MoS_2_ memtransistors to mimic neuromodulated heterosynaptic plasticity.

Modulatory‐induced homosynaptic (M‐) plasticity (i.e., in the absence of *V*_pre_) was investigated for the same BC TCTA/MoS_2_ memtransistors. As shown in Figure 4c (M‐LTP), the PSC gradually increased during potentiation (*V*_mod_ = −40 V with 100 pulses) and sequentially decreased during depression (*V*_mod_ = 30 V with 100 pulses). In addition, the M‐STDP (Figure 4d) demonstrated polarity‐dependent strengthening (with *V*_mod_ = −40 V) and weakening (with *V*_mod_ = 30 V). Notably, the *w* in M‐STDP presented characteristic decay curves with *τ*_+_ = 100 ms for potentiation (Δ*t* > 0) and *τ*_−_ = 60 ms for depression (Δ*t* < 0). These results are strongly correlated with the gate controllability in H‐plasticity, supporting the polarity‐dependent memristive synaptic characteristics of the BC TCTA/MoS_2_ memtransistors. The similar gate‐modulated synaptic behaviors were observed in different batches of the BC TCTA/MoS_2_ memtransistors, indicating reproducibility (Figure S13).

## 3 Discussion

Previous studies on organic/inorganic memristors with vertical or planar structures have focused on hybrid effects and photo‐induced activation, including the incorporation of interface modification and defect engineering to realize neuromorphic computing [53, 54, 55, 56, 57, 58]. Although these devices exhibit resistive‐switching‐based memory characteristics, few studies have quantitatively demonstrated synaptic weight modulation or analyzed STDP behavior. In contrast, three‐terminal memtransistor architectures based on FET configurations have recently garnered attention as a promising route toward heterosynaptic modulation, enabling direct tuning of the channel conductance via gate bias. Wang et al. reported a multi‐functional synaptic transistor based on perylene‐3,4,9,10‐tetracarboxylic dianhydride (PTCDA) / MoS_2_ hybrid heterojunction, with flexible tunability of STP and LTP [59]. Photonic synaptic transistors based on dinaphtho[2,3‐*b*:2’,3’‐*f*]thieno[3,2‐*b*]thiophene (DNTT) / MoS_2_ organic semiconductor heterojunction demonstrated a low energy consumption of 0.4 fJ per synaptic event with ultraweak light intensity of 40 nW cm^−2^ for neuromorphic computing [60]. However, systematic quantification of STDP remains insufficient even in these multi‐terminal structures, which often require intricate fabrication steps, such as the insertion of ferroelectric layers or the induction of photo‐assisted ion migration, to realize synaptic functionality [54, 55].

The BC TCTA/MoS_2_ memtransistors presented in this study address these challenges. Without additional defect‐generation or surface‐modification processes, our BC TCTA/MoS_2_ memtransistors exhibited STDP behavior that could be quantitatively analyzed (*τ*_+_ = 100 ms, *τ*_−_ = 60 ms) and reproducible synaptic weight modulation solely under gate‐pulse operation. Moreover, the memtransistors were fabricated with a simple bottom‐contact heterojunction structure composed of a π‐conjugated organic semiconductor (TCTA) and a 2D inorganic semiconductor (MoS_2_), without using h‐BN insulating layers or post‐treatment steps, while maintaining stable electrical characteristics. This reproducible heterojunction configuration demonstrated consistent memristive switching (on/off ≈ 10^2^) and stable heterosynaptic plasticity across different batches of devices. Therefore, the proposed architecture effectively bridges the gap between non‐tunable two‐terminal memristors and complex multi‐terminal synaptic transistors, offering a scalable and reliable platform for next‐generation neuromorphic electronic systems. For future work of our BC TCTA/MoS_2_ memtransistors, to reduce the power consumption and the degradation of dielectric layer over cycles in memtransistors, their working gate voltage should be reduced by optimizing the energy‐band offset of TCTA/MoS_2_ heterojunctions to increase charge transfer efficiency, integrating high‐κ ‐ dielectrics (e.g., HfO_2_, ZrSiO_4_, ZrO_2_, Al_2_O_3_) and/or ferroelectrics to enhance gate electric field coupling, or thinning the active layer to reduce resistance and lower voltage[Table advs73598-tbl-0001] requirements.

**TABLE 1 advs73598-tbl-0001:** Comparison of key parameters of the BC TCTA/MoS_2_ memtransistor with other reported memtransistors and memristors.

Device	Active layer	Switching ratio	Operating voltage	Potentiation/Depression time	Refs.
Memtransistor	TCTA/MoS_2_ HS	∼10^2^	*V*_G_ = −40–30 V *V*_D_ = −6–6 V	100 ms/60 ms	This work
	Polycrystalline MoS_2_	∼10^2^	*V*_G_ = −50–50 V *V*_D_ = −80–80 V	2 ms/6 ms	[1]
	MAPbI_3‐x_Br_x_/IDT‐BT	∼10^2^	*V*_G_ = −80–0 V *V*_D_ = −80–80 V	LTP/LTD observed	[53]
	Rubrene/CulnP_2_S	—	*V*_G_ = −60–60 V *V*_D_ = −80–0 V	—	[54]
	PTCDA/MoS_2_	—	*V*_G_ = −20 to −12 V *V*_D_ = 0.1–1 V	STP/LTP observed	[59]
	DNTT/MoS_2_		*V*_D_ = 0.001–0.2 V *(No gate voltage used)*	STP/LTP Observed	[60]
Synaptic transistor	Organic semiconductor / CuInSe_2_ QDs	—	*V*_G_ = −40–10 V *V*_D_ = −30–0 V	LTP/LTD, STDP observed	[55]
Memristor	MoS_2_	4–6	0–60 V	—	[37]
	MoS_2_	∼10^3^	−5–5 V	LTP/LTD observed	[56]
	Al‐HQ hybrid/Al_2_O_3_	∼10^3^	*V*_set_ = 1 V *V*_reset_ = −0.7 V	2 ms/6 ms	[57]
	ZnO nanorods/PMMA	—	*V*_set_ = 3 V *V*_reset_ = −3 V	LTP/LTD observed	[58]

Recent reviews on future neuromorphic applications of memresistive devices provide potential solutions for efficient brain‐inspired computation with memristive implementations: as accelerators for deep learning and artificial intelligence and as building blocks for spiking neural networks and in‐sensor computing [10, 61, 62, 63].

## 4 Conclusion

Novel memtransistors incorporating TCTA and MoS_2_ HSs as the active layer were designed using type‐II energy‐band engineering and a BC architecture. Memristive switching was achieved via gate‐pulse modulation. Memristor characteristics with SET and RESET hysteresis in the *V*_G_ < *V*_th_ regime could be interpreted through the trap‐related SCLC model. The devices successfully mimicked synaptic functions via tunable and non‐volatile changes in PSC in response to electrical stimuli, with the drain, source, and gate terminals functioning as the pre‐neuron, post‐neuron, and modulatory neuron in biological neuron systems, respectively. Measured current vs. voltage transfer and output characteristics at various gate biases/pulses clearly demonstrated polarity‐dependent synaptic behavior, consistent with H‐LTP, highlighting the capability of emulating neuromodulated heterosynaptic plasticity. This work highlights the potential of the designed organic/inorganic‐semiconducting HSs for memtransistors in promoting the implementation of reliable neuromorphic electronics.

## 5 Experimental Section

### 5.1 Device Fabrication

TCTA powder (purity > 98.0%) was purchased from Ossila and used without further purification. SiO_2_/Si substrates were sonicated for 5 min in acetone, cleaned with isopropyl alcohol, and treated with ozone for 30 min. The TC and BC FETs were fabricated under identical conditions, except for their stacking sequences. For the TC FETs, few‐layer (∼10 nm) MoS_2_ (HQ Graphene) flakes were mechanically exfoliated and transferred onto the ozone‐treated SiO_2_/Si substrates. Subsequently, Au/Ti (50/5 nm) electrodes were deposited onto the MoS_2_ layer via e‐beam lithography with conventional thermal evaporation. For the BC FETs, the electrodes were patterned first, and the exfoliated MoS_2_ flakes were transferred onto them later. To prepare the p–n HS, TCTA with a thickness of 30 nm was deposited onto the TC and BC pristine MoS_2_ FETs using a custom‐built organic molecular beam deposition equipment (DAEKI High‐Tech.) at a deposition rate of 1 nm min^−1^ under ∼10^−6^ Torr.

### 5.2 Measurements

Electrical measurements were performed in a vacuum probe station at 295 K under ∼60 mTorr using source‐measure units (237 and 2634B; Keithley). The drain voltage was swept from −3 to 3 V, and the gate voltage was varied from −30 to 30 V. Synaptic behaviors including LTP and STDP were characterized using custom Python programs based on the built‐in commands of the 2634B unit, along with an oscilloscope (TDS 2022C; Tektronix). A read pulse of −3 V was applied to the drain. For pre‐synaptic pulses, +6 and −6 V were applied to the drain. For modulatory pulses, −40, 0, and 30 V were applied to the gate. The pulse width was fixed at 5 ms, and the interval was varied between 5 and 50 ms. Details of the synaptic function measurement conditions and sequences are presented in the SI. UPS was performed using a Nexsa system (Thermo Fisher Scientific) with an excitation source of He I (21.22 eV) at the Korea Institute of Science and Technology. UV–visible (UV–vis) absorbance spectra were measured using a UV/vis spectrometer (8453; Agilent).

## Conflicts of Interest

The authors declare no conflicts of interest.

## Supporting information




**Supporting file**: advs73598‐sup‐0001‐SuppMat.docx

## Data Availability

The data that support the findings of this study are available from the corresponding author upon reasonable request.

## References

[1] V. K. Sangwan , H.‐S. Lee , H. Bergeron , et al., “Multi‐Terminal Memtransistors from Polycrystalline Monolayer Molybdenum Disulfide,” Nature 554 (2018): 500–504, 10.1038/nature25747. 29469093

[2] T. You , M. Zhao , Z. Fan , and C. Ju , “Emerging Memtransistors for Neuromorphic System Applications: A Review,” Sensors 23 (2023): 5413, 10.3390/s23125413. 37420582 PMC10302604

[3] W. Huh , D. Lee , S. Jang , et al., “Heterosynaptic MoS_2_ Memtransistors Emulating Biological Neuromodulation for Energy‐Efficient Neuromorphic Electronics,” Advanced Materials 35 (2023): 2211525, 10.1002/adma.202211525. 36930856

[4] J. Bae , J. Won , and W. Shim , “The Rise of Memtransistors for Neuromorphic Hardware and In‐Memory Computing,” Nano Energy 126 (2024): 109646, 10.1016/j.nanoen.2024.109646.

[5] L. Chua , “Memristor‐The Missing Circuit Element,” IEEE Transactions on Circuit Theory 18 (1971): 507–516, 10.1109/tct.1971.1083337.

[6] L. O. Chua and S. M. Kang , “Memristive Devices and Systems,” Proceedings of the IEEE 64 (1976): 209–223, 10.1109/proc.1976.10092.

[7] D. B. Strukov , G. S. Snider , D. R. Stewart , and R. S. Williams , “The Missing Memristor Found,” Nature 453 (2008): 80–83, 10.1038/nature06932. 18451858

[8] J. J. Yang , D. B. Strukov , and D. R. Stewart , “Memristive Devices for Computing,” Nature Nanotechnology 8 (2013): 13–24, 10.1038/nnano.2012.240. 23269430

[9] D. S. Jeong , K. M. Kim , S. Kim , B. J. Choi , and C. S. Hwang , “Memristors for Energy‐Efficient New Computing Paradigms,” Advanced Electronic Materials 2, (2016): 1600090, 10.1002/aelm.201600090.

[10] Q. Xia and J. J. Yang , “Memristive Crossbar Arrays for Brain‐Inspired Computing,” Nature Materials 18 (2019): 309–323, 10.1038/s41563-019-0291-x. 30894760

[11] R. Waser , R. Dittmann , G. Staikov , and K. Szot , “Redox‐Based Resistive Switching Memories—Nanoionic Mechanisms, Prospects, and Challenges,” Advanced Materials 21 (2009): 2632–2640, 10.1002/adma.200900375. 36751064

[12] L. Chua , “Resistance Switching Memories Are Memristors,” Applied Physics A 102 (2011): 765–783, 10.1007/s00339-011-6264-9.

[13] D. Kuzum , S. Yu , and H. S. P. Wong , “Synaptic electronics: Materials, devices and applications,” Nanotechnology 24 (2013): 382001, 10.1088/0957-4484/24/38/382001. 23999572

[14] M. A. Zidan , J. P. Strachan , and W. D. Lu , “The Future of Electronics Based on Memristive Systems,” Nature Electronics 1 (2018): 22–29, 10.1038/s41928-017-0006-8.

[15] Z. Li , W. Tang , B. Zhang , R. Yang , and X. Miao , “Emerging Memristive Neurons for Neuromorphic Computing and Sensing,” Science and Technology of Advanced Materials 24 (2023): 2188878, 10.1080/14686996.2023.2188878. 37090846 PMC10120469

[16] I. M. Kipelkin , S. A. Gerasimova , A. I. Belov , et al., “Memristor‐Based Model of Neuronal Excitability and Synaptic Potentiation,” Frontiers in Neuroscience 18 (2024): 1456386, 10.3389/fnins.2024.1456386. 39624404 PMC11609164

[17] R. Yang , H.‐M. Huang , and X. Guo , “Memristive Synapses and Neurons for Bioinspired Computing,” Advanced Electronic Materials 5 (2019): 1900287, 10.1002/aelm.201900287.

[18] H. Peng , L. Gan , and X. Guo , “Memristor‐based spiking neural networks: Cooperative development of neural network architecture/algorithms and memristors,” Chip 3 (2024): 100093, 10.1016/j.chip.2024.100093.

[19] S.‐O. Park , H. Jeong , J. Park , J. Bae , and S. Choi , “Experimental Demonstration of Highly Reliable Dynamic Memristor for Artificial Neuron and Neuromorphic Computing,” Nature Communications 13 (2022): 2888, 10.1038/s41467-022-30539-6. PMC9166790 35660724

[20] J. J. Yang , M. D. Pickett , X. Li , D. A. Ohlberg , D. R. Stewart , and R. S. Williams , “Memristive Switching Mechanism for Metal/Oxide/Metal Nanodevices,” Nature Nanotechnology 3 (2008): 429–433, 10.1038/nnano.2008.160. 18654568

[21] G.‐S. Park , Y. B. Kim , S. Y. Park , et al., “In Situ Observation of Filamentary Conducting Channels in an Asymmetric Ta2O5−x/TaO2−x Bilayer Structure,” Nature Communications 4 (2013): 2382, 10.1038/ncomms3382. 24008898

[22] R. Leal Martir , M. José Sánchez , M. Aguirre , et al., “Oxygen Vacancy Dynamics in Pt/TiOx/TaOy/Pt Memristors: Exchange with the Environment and Internal Electromigration,” Nanotechnology 34 (2022): 095202, 10.1088/1361-6528/aca597. 36541534

[23] A. Wedig , M. Luebben , D.‐Y. Cho , et al., “Nanoscale Cation Motion in TaOx, HfOx and TiOx Memristive Systems,” Nature Nanotechnology 11 (2016): 67–72, 10.1038/nnano.2015.221. 26414197

[24] M. Ismail , M. Rasheed , C. Mahata , M. Kang , and S. Kim , “Mimicking Biological Synapses with a‐HfSiOx‐Based Memristor: Implications for Artificial Intelligence and Memory Applications,” Nano Convergence 10 (2023): 33, 10.1186/s40580-023-00380-8. 37428275 PMC10333172

[25] M. Praveen , A. K. Nishad , and V. K. Nishad , “Nanoscale Ni/Mo/MoO3/Ni Memristor for Synaptic Applications,” Electronics Letters 60 (2024): 13131, 10.1049/ell2.13131.

[26] M. Wang , S. Cai , C. Pan , et al., “Robust Memristors Based on Layered Two‐Dimensional Materials,” Nature Electronics 1 (2018): 130–134, 10.1038/s41928-018-0021-4.

[27] M. Kumar , D. K. Ban , S. M. Kim , J. Kim , and C. P. Wong , “Vertically Aligned WS 2 Layers for High‐Performing Memristors and Artificial Synapses,” Advanced Electronic Materials 5 (2019): 1900467, 10.1002/aelm.201900467.

[28] X. Yan , K. Wang , J. Zhao , et al., “A New Memristor With 2D Ti 3 C 2 T x MXene Flakes as an Artificial Bio‐Synapse,” Small 15 (2019): 1900107, 10.1002/smll.201900107. 31066210

[29] J. Xie , M. N. Patoary , M. A. Rahman Laskar , et al., “Quantum Conductance in Vertical Hexagonal Boron Nitride Memristors With Graphene‐Edge Contacts,” Nano Letters 24 (2024): 2473–2479, 10.1021/acs.nanolett.3c04057. 38252466

[30] A. A. Bessonov , M. N. Kirikova , D. I. Petukhov , M. Allen , T. Ryhanen , and M. J. Bailey , “Layered Memristive and Memcapacitive Switches for Printable Electronics,” Nature Materials 14 (2015): 199–204, 10.1038/nmat4135. 25384168

[31] W. Huh , S. Jang , J. Y. Lee , et al., “Synaptic Barristor Based on Phase‐Engineered 2D Heterostructures,” Advanced Materials 30 (2018): 1801447, 10.1002/adma.201801447. 30015988

[32] X. Yan , Q. Zhao , A. P. Chen , et al., “Vacancy‐Induced Synaptic Behavior in 2D WS 2 Nanosheet–Based Memristor for Low‐Power Neuromorphic Computing,” Small 15 (2019): 1901423, 10.1002/smll.201901423. 31045332

[33] Y. Shi , X. Liang , B. Yuan , et al., “Electronic Synapses Made of Layered Two‐Dimensional Materials,” Nature Electronics 1 (2018): 458–465, 10.1038/s41928-018-0118-9.

[34] R. Xu , H. Jang , M.‐H. Lee , et al., “Vertical MoS2 Double‐Layer Memristor with Electrochemical Metallization as an Atomic‐Scale Synapse with Switching Thresholds Approaching 100 mV,” Nano Letters 19 (2019): 2411–2417, 10.1021/acs.nanolett.8b05140. 30896171

[35] P. Cheng , K. Sun , and Y. H. Hu , “Memristive Behavior and Ideal Memristor of 1T Phase MoS2 Nanosheets,” Nano Letters 16 (2016): 572–577, 10.1021/acs.nanolett.5b04260. 26654683

[36] F. Zhang , H. Zhang , S. Krylyuk , et al., “Electric‐Field Induced Structural Transition in Vertical MoTe2‐ and Mo1–xWxTe2‐Based Resistive Memories,” Nature Materials 18 (2019): 55–61, 10.1038/s41563-018-0234-y. 30542093

[37] R. Ge , X. Wu , M. Kim , et al., “Atomristor: Nonvolatile Resistance Switching in Atomic Sheets of Transition Metal Dichalcogenides,” Nano Letters 18 (2018): 434–441, 10.1021/acs.nanolett.7b04342. 29236504

[38] Z. Dong , Q. Hua , J. Xi , et al., “Ultrafast and Low‐Power 2D Bi2O2Se Memristors for Neuromorphic Computing Applications,” Nano Letters 23 (2023): 3842–3848, 10.1021/acs.nanolett.3c00322. 37093653

[39] A. C. Khot , K. A. Nirmal , T. D. Dongale , and T. G. Kim , “GeTe/MoTe2 Van der Waals Heterostructures: Enabling Ultralow Voltage Memristors for Nonvolatile Memory and Neuromorphic Computing Applications,” Small 20 (2024): 2400791, 10.1002/smll.202400791. 38874088

[40] V. K. Sangwan , D. Jariwala , I. S. Kim , et al., “Gate‐tunable memristive phenomena mediated by grain boundaries in single‐layer MoS2,” Nature Nanotechnology 10 (2015): 403–408, 10.1038/nnano.2015.56. 25849785

[41] M. Raoufi , S. Chandrabose , R. Wang , et al., “Influence of the Energy Level Alignment on Charge Transfer and Recombination at the Monolayer‐MoS2/Organic Hybrid Interface,” The Journal of Physical Chemistry C 127 (2023): 5866–5874, 10.1021/acs.jpcc.2c08186.

[42] A. Rose , “Space‐Charge‐Limited Currents in Solids,” Physical Review 97 (1955): 1538–1544, 10.1103/PhysRev.97.1538.

[43] J. A. Geurst , “Theory of Space‐Charge‐Limited Currents in Thin Semiconductor Layers,” Physica Status Solidi (b) 15 (2006): 107–118, 10.1002/pssb.19660150108.

[44] N. F. Mott and R. W. Gurney , Electronic Processes in Ionic Crystals 2nd ed., International Series of Monographs on Physics (Clarendon Press, 1948).

[45] P. Mark and W. Helfrich , “Space‐Charge‐Limited Currents in Organic Crystals,” Journal of Applied Physics 33 (1962): 205–215, 10.1063/1.1728487.

[46] C.‐H. Lee , G.‐H. Lee , A. M. van der Zande , et al., “Atomically thin p–n junctions with Van Der Waals heterointerfaces,” Nature Nanotechnology 9 (2014): 676–681, 10.1038/nnano.2014.150. 25108809

[47] C. J. Park , H. J. Park , J. Y. Lee , J. Kim , C. H. Lee , and J. Joo , “Photovoltaic Field‐Effect Transistors Using a MoS2 and Organic Rubrene van der Waals Hybrid,” ACS Applied Materials & Interfaces 10 (2018): 29848–29856, 10.1021/acsami.8b11559. 30091581

[48] S. M. Sze and K. K. Ng , Physics of Semiconductor Devices, 3rd ed. (Wiley, 2006).

[49] L. F. Abbott and S. B. Nelson , “Synaptic Plasticity: Taming the Beast,” Nature Neuroscience 3 (2000): 1178–1183, 10.1038/81453. 11127835

[50] N. Du , M. Kiani , C. G. Mayr , et al., “Single Pairing Spike‐Timing Dependent Plasticity in BiFeO_3_ Memristors with a Time Window of 25 ms to 125 µs,” Frontiers in Neuroscience 9 (2015): 227, 10.3389/fnins.2015.00227. 26175666 PMC4485154

[51] S. Song , K. D. Miller , and L. F. Abbott , “Competitive Hebbian Learning Through Spike‐Timing‐Dependent Synaptic Plasticity,” Nature Neuroscience 3 (2000): 919–926, 10.1038/78829. 10966623

[52] S. H. Jo , T. Chang , I. Ebong , B. B. Bhadviya , P. Mazumder , and W. Lu , “Nanoscale Memristor Device as Synapse in Neuromorphic Systems,” Nano Letters 10 (2010): 1297–1301, 10.1021/nl904092h. 20192230

[53] C. Ma , H. Chen , E. Yengel , et al., “Printed Memtransistor Utilizing a Hybrid Perovskite/Organic Heterojunction Channel,” ACS Applied Materials & Interfaces 13 (2021): 51592–51601, 10.1021/acsami.1c08583. 34696578

[54] K. Pei , “Rubrene/CuInP2S6 Organic/Inorganic Heterojunctions for Ferroelectric Organic Transistor Memory Devices,” Surfaces and Interfaces 38 (2023): 102801, 10.1016/j.surfin.2023.102801.

[55] J. Zhang , Z. Guo , T. Sun , et al., “Energy‐Efficient Organic Photoelectric Synaptic Transistors with Environment‐Friendly CuInSe 2 Quantum Dots for Broadband Neuromorphic Computing,” SmartMat 5 (2024): 1246, 10.1002/smm2.1246.

[56] E. Lee , J. Kim , J. Park , et al., “Realizing Electronic Synapses by Defect Engineering in Polycrystalline Two‐Dimensional MoS2 for Neuromorphic Computing,” ACS Applied Materials & Interfaces 15 (2023): 15839–15848, 10.1021/acsami.2c21688. 36919898

[57] C. Liu , Y.‐J. Ma , S. Sun , et al., “A Multilevel Resistive Switching Memristor based on Flexible Organic–Inorganic Hybrid Film with Recognition Function,” Journal of Physics D: Applied Physics 58 (2025): 025101, 10.1088/1361-6463/ad835e.

[58] A. H. Jaafar and N. T. Kemp , “Light‐Mediated Multilevel Neuromorphic Switching in a Hybrid Organic–Inorganic Memristor,” ACS Omega 9 (2024): 51641–51650, 10.1021/acsomega.4c09401. 39758653 PMC11696397

[59] S. Wang , C. Chen , Z. Yu , et al., “A MoS2/PTCDA Hybrid Heterojunction Synapse With Efficient Photoelectric Dual Modulation and Versatility,” Advanced Materials 31 (2019): 1806227, 10.1002/adma.201806227. 30485567

[60] J. Wang , B. Yang , S. Dai , et al., “Weak Light‐Stimulated Synaptic Transistors Based on MoS 2 /Organic Semiconductor Heterojunction for Neuromorphic Computing,” Advanced Materials Technologies 8 (2023): 2300449, 10.1002/admt.202300449.

[61] H. Li , Z. Wang , Y. Zhao , D. Geng , D. Ji , and W. Hu , “Recent Progress in 2‐Dimensional Organic–Inorganic Heterojunction Optoelectronic Devices,” Advanced Devices & Instrumentation 6 (2025): 0069, 10.34133/adi.0069.

[62] M.‐K. Song , J.‐H. Kang , X. Zhang , et al., “Recent Advances and Future Prospects for Memristive Materials, Devices, and Systems,” ACS Nano 17 (2023): 11994–12023, 10.1021/acsnano.3c03505. 37382380

[63] H. Zhou , S. Li , K. W. Ang , and Y. W. Zhang , “Recent Advances in In‐Memory Computing: Exploring Memristor and Memtransistor Arrays With 2D Materials,” Nano‐Micro Letters 16 (2024): 121, 10.1007/s40820-024-01335-2. 38372805 PMC10876512

