# Supplementary material for: Heterosynaptic Memtransistors Based on Switching Operation Mechanism Using Designed Organic/Inorganic Heterostructures for Neuromorphic Electronics
Source: Adv Sci (Weinh). 2026 Jan 5;13(14):e17149. doi: 10.1002/advs.202517149 (PMC12970180; doi:10.1002/advs.202517149)
Supplement: Supplementary file 1 — Supporting file: advs73598‐sup‐0001‐SuppMat.docx [file ADVS-13-e17149-s001.docx]

Supporting Information

Heterosynaptic Memtransistors Based on Switching Operation Mechanism Using Designed Organic/Inorganic Heterostructures for Neuromorphic Electronics

Taek Joon Kim, Hye Lim Jeong, Sang Wook Song, Dayeong Kwon, Sang-hun Lee, and Jinsoo Joo^*^

**S-1. Materials and Interface characteristics**

UPS and UV–vis absorption spectra of TCTA are shown in Figure S1.


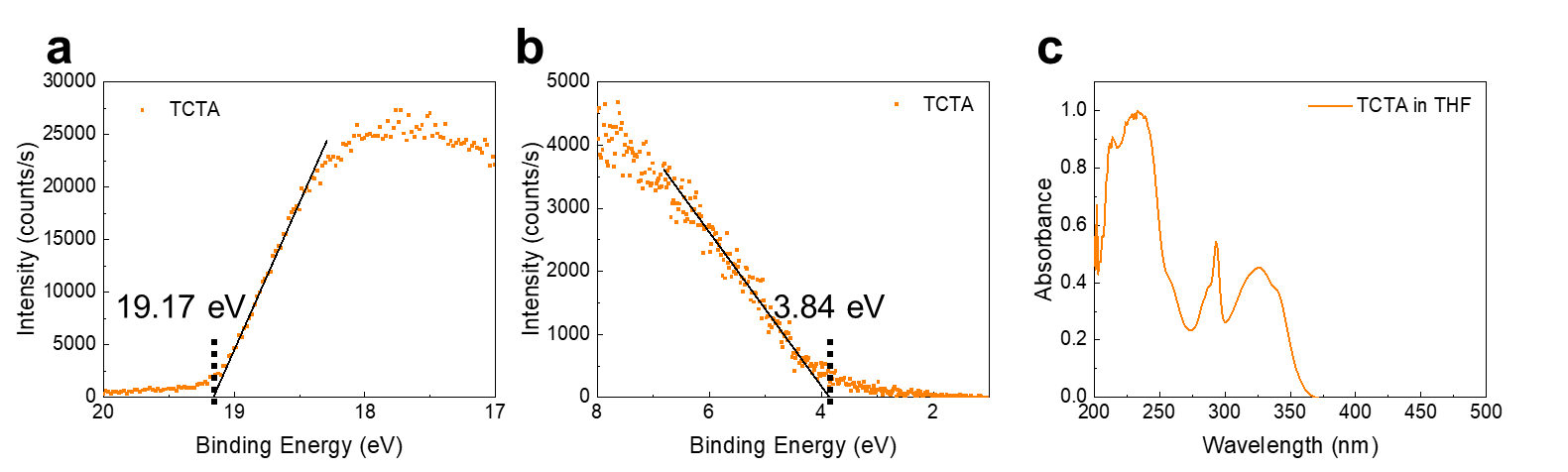


**Figure S1.** UPS and UV–vis absorption spectra of TCTA. a) Secondary electron cutoff b) and valence band regions in UPS spectra for TCTA. c) UV–vis absorption spectrum of TCTA.

Figure S2 shows the absorbance of TCTA in THF solution and the PL spectrum of the TCTA thin film. The absorption edge of TCTA was observed at 350 nm. The PL peaks of TCTA thin film, observed at 400 nm and 540 nm, corresponded to the Frenkel exciton (XF) and excimer (XM), respectively.


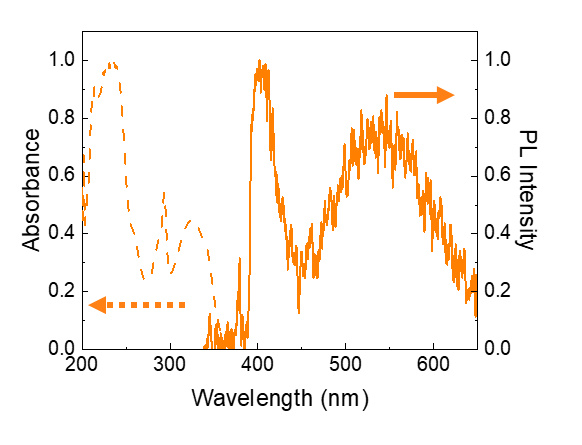


**Figure S2.** Absorbance (dotted curve) of TCTA in THF solution and PL spectra (solid curve) of TCTA thin film.

Figure S3 shows the height (thickness) profile of a few layers of MoS_2_ for 7 different samples. The average thickness of a few layers of MoS_2_ for this study was 3.46 nm.


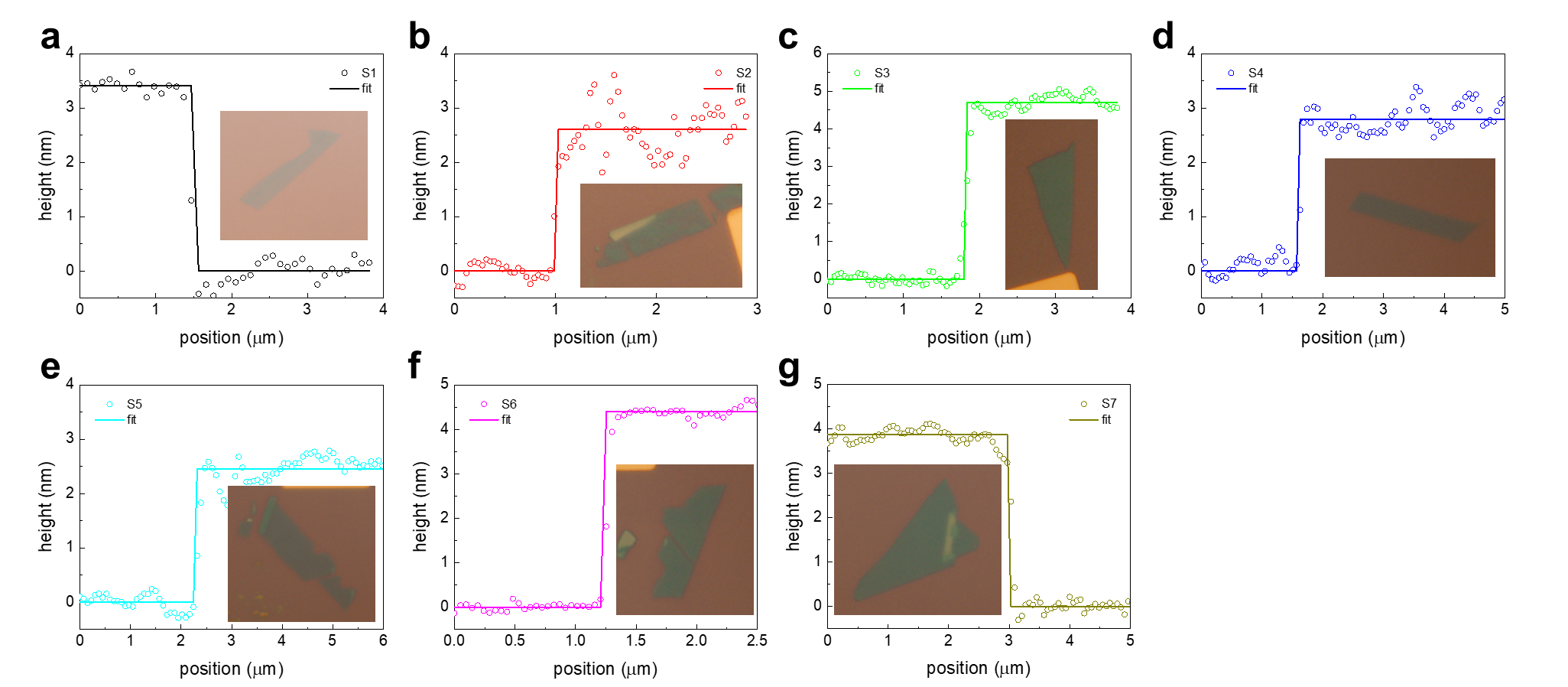


**Figure S3.** Thickness profiles using AFM and optical images (insets) of a few layers of MoS_2_ for 7 different samples.

**Table S1.** Thicknesses of a few layers of MoS_2_ obtained from Figure S3 of AFM images.

| Sample | S1 | S2 | S3 | S4 | S5 | S6 | S7 |
| --- | --- | --- | --- | --- | --- | --- | --- |
| Thickness  (nm) | 3.41 | 2.60 | 4.70 | 2.78 | 2.45 | 4.40 | 3.87 |

Figure S4a shows the energy band diagram of the TCTA/MoS_2_ HS, obtained from UPS, absorption, and XPS experiments. Figure S4b presents the XPS spectra of pristine MoS_2_ and the TCTA/MoS_2_ HS. The Mo 3d_5/2_ peak of pristine MoS_2_ was observed at 229.37 eV. After hybridization with TCTA, the peak shifted slightly to a higher binding energy (229.57 eV), attributable to sulfur vacancy passivation^[S1]^ and/or hole transfer from MoS_2_ to TCTA.^[S2]^ This result suggests that the HOMO level of TCTA is higher than the VBM of MoS_2_, consistent with our band diagram (Figure 1b).


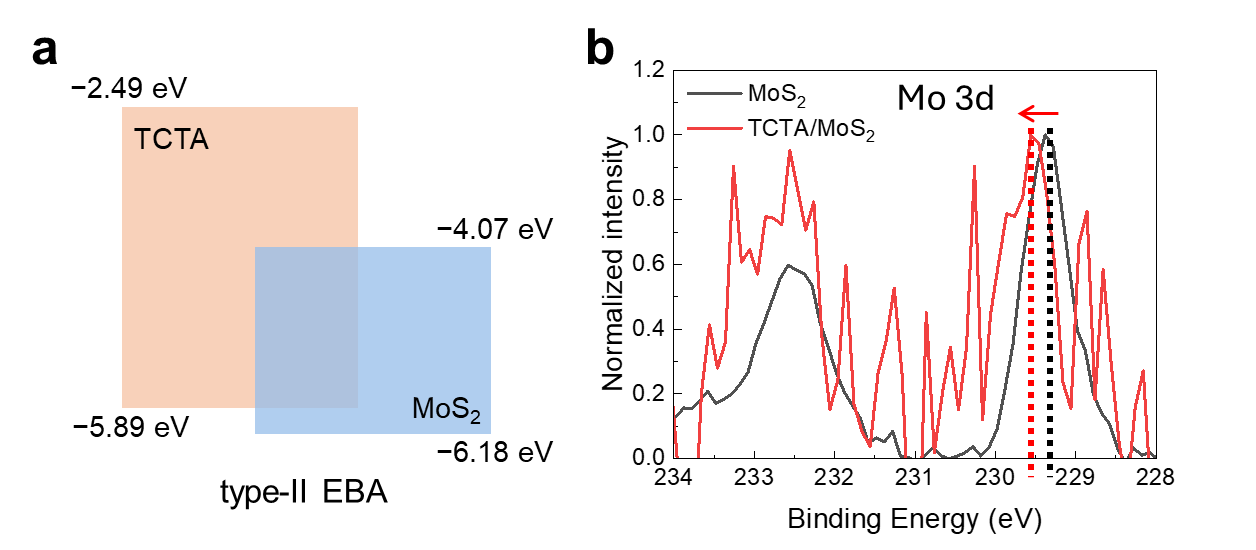


**Figure S4.** a) Energy band diagram of the TCTA/MoS_2_ HS. b) XPS spectra of the pristine MoS_2_ and TCTA/MoS_2_ HS.

**S-2. Charge transport mechanisms of top-contact (TC) and bottom-contact (BC) TCTA/MoS_2_** **FETs.**

In top-contact (TC) MoS_2_-based field-effect transistors (FETs), electrons accumulated at the bottom of the MoS_2_ layer at *V*_G_ > *V*_th_ because of the n-type characteristics of the MoS_2_ layer, as illustrated in Figure S5a. This behavior was confirmed from the transfer (*I*_D_–*V*_G_) and output (*I*_D_–*V*_D_) characteristic curves (Figure S5b–f). The transfer curves of the TC TCTA/MoS_2_ FET (solid red markers in Figure S5b) revealed typical n-type behavior, similar to that of the TC MoS_2_ FET (open black markers in Figure S5b). The threshold voltages (*V*_th_) were estimated to be approximately −30 V for the pristine MoS_2_ and −20 V for the TCTA/MoS_2_ FET. The output characteristic curves of both TC MoS_2_ and TCTA/MoS_2_ FETs showed non-hysteresis characteristics at negative *V*_G_ (Figure 5c–f). These results indicate the complete lack or negligible contribution from the TCTA layer to the current transport in the TC FET, likely owing to the high resistance of TCTA.

In bottom-contact (BC) FETs, the charge transport mechanism varied depending on *V*_G_. For *V*_G_ > *V*_th_, the major current channel was formed at the bottom of MoS_2_, analogous to that of the TC FETs (Figure S6b). As *V*_G_ became increasingly negative, the electrons in MoS_2_ accumulated at the top of the layer, while the holes in TCTA accumulated at the bottom, that is, both electrons and holes accumulated at the heterointerface (Figure S6a). In this regime, the concave shape of the MoS_2_ channel induced charge accumulation within a limited space (space-charge region in Figure S6a). For BC TCTA/MoS_2_ memtransistors, the high carrier concentration contributed to the enhancement of interfacial recombination rates via trap-intermediated Shockley–Read–Hall and Langevin processes (tunneling and recombination regions in Figure S6a), resulting in the formation of a current channel through the MoS_2_–TCTA–MoS_2_ n–p–n junction. As a result, the heterostructure with BC architecture exhibited different conduction mechanisms depending on *V*_G_.

To explore gate tunability, the output characteristic curves (*I*_D_–*V*_D_) of the BC TCTA/MoS_2_ memtransistor were measured at various *V*_G_ (−30, −20, −15, −10, 0, 10, 15, 20, and 30 V), as shown in Figure 2a and Figure S7.


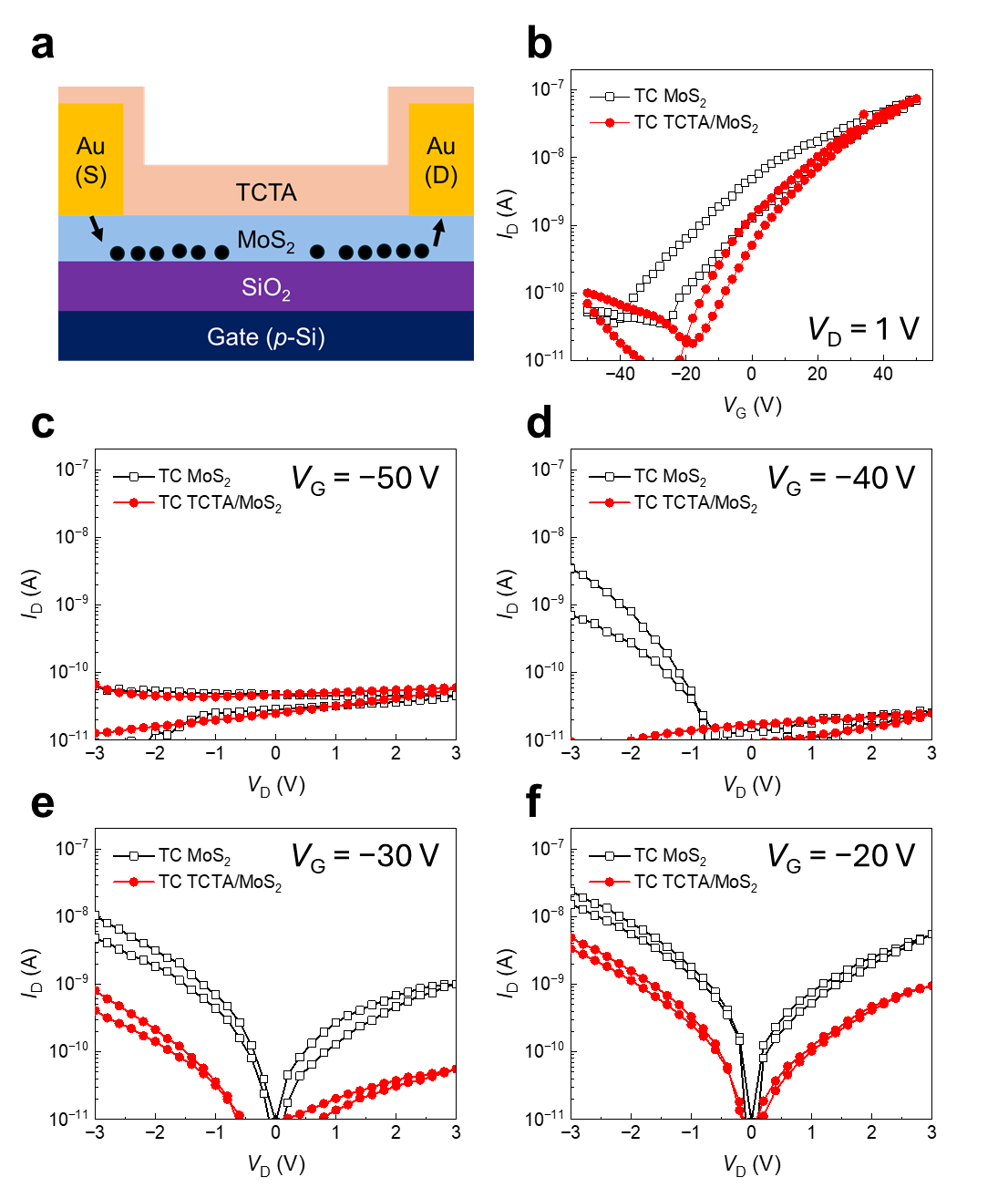


**Figure S5.** Top-contact (TC) MoS_2_-based FETs. a) Schematic of the charge transport mechanism in TC TCTA/MoS_2_ FET at *V*_G_ > *V*_th_. b) Transfer characteristic curves (*I*_D_–*V*_G_) of the TC MoS_2_-based FETs at *V*_D_ = 1 V before (open black markers) and after (solid red markers) hybridization with TCTA. Output characteristic curves (*I*_D_–*V*_D_) of the TC MoS_2_-based FETs at *V*_G_ = c) −50 V, d) −40 V, e) −30 V, and f) −20 V, before (open black markers) and after (solid red markers) hybridization with TCTA.


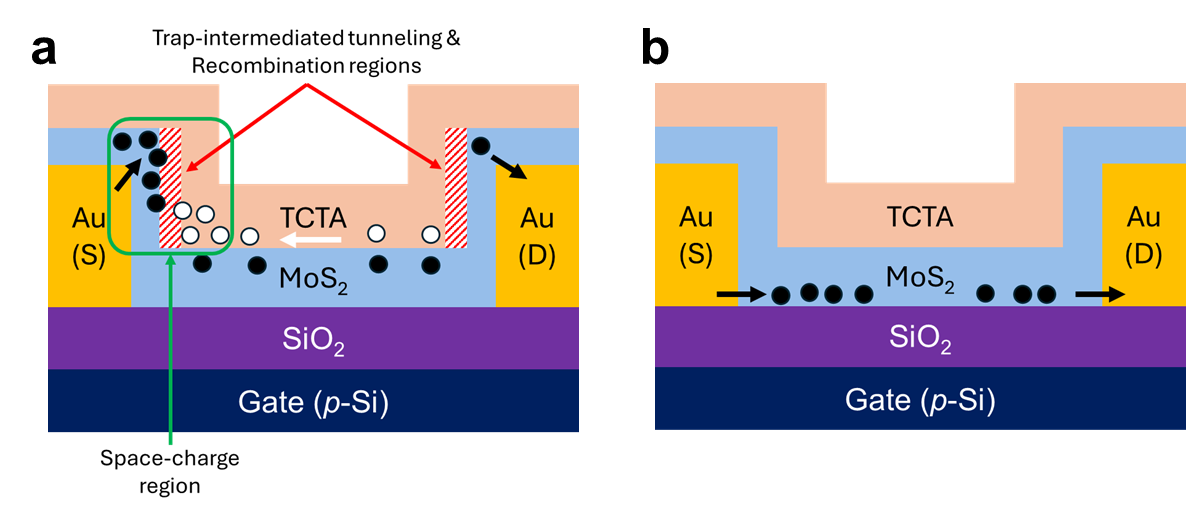


**Figure S6.** Charge transport mechanism in BC TCTA/MoS_2_ memtransistors. Schematic of charge transport mechanisms in BC TCTA/MoS_2_ memtransistors with *V*_G_ a) less than and b) more than *V*_th_.

Using a higher *V*_D_ = 2 V, transfer characteristic curves (*I*_D_–*V*_G_) similar to those in Figure 1d were observed (Figure S7a). The hysteretic output characteristic curves (*I*_D_–*V*_D_) were observed for the BC TCTA/MoS_2_ FET with negative gate voltages (–20 V and –30 V), as shown in Figures S7b–h. As *V*_G_ increased from −30 V to −10 V (Figures S7b to d), resistive switching behavior was observed in the BC TCTA/MoS_2_ memtransistor with decreasing switching ratio. From −10 V to +30 V (Figures S7d-h), hysteresis was not clearly observed. Under negative *V*_G_, electrons and holes accumulated at the interface between the TCTA and MoS_2_ heterojunction (Figure S6a), whereas under positive *V*_G_, electrons, as major charge carriers, dominantly flowed in the MoS_2_ layer (Figure S6b).


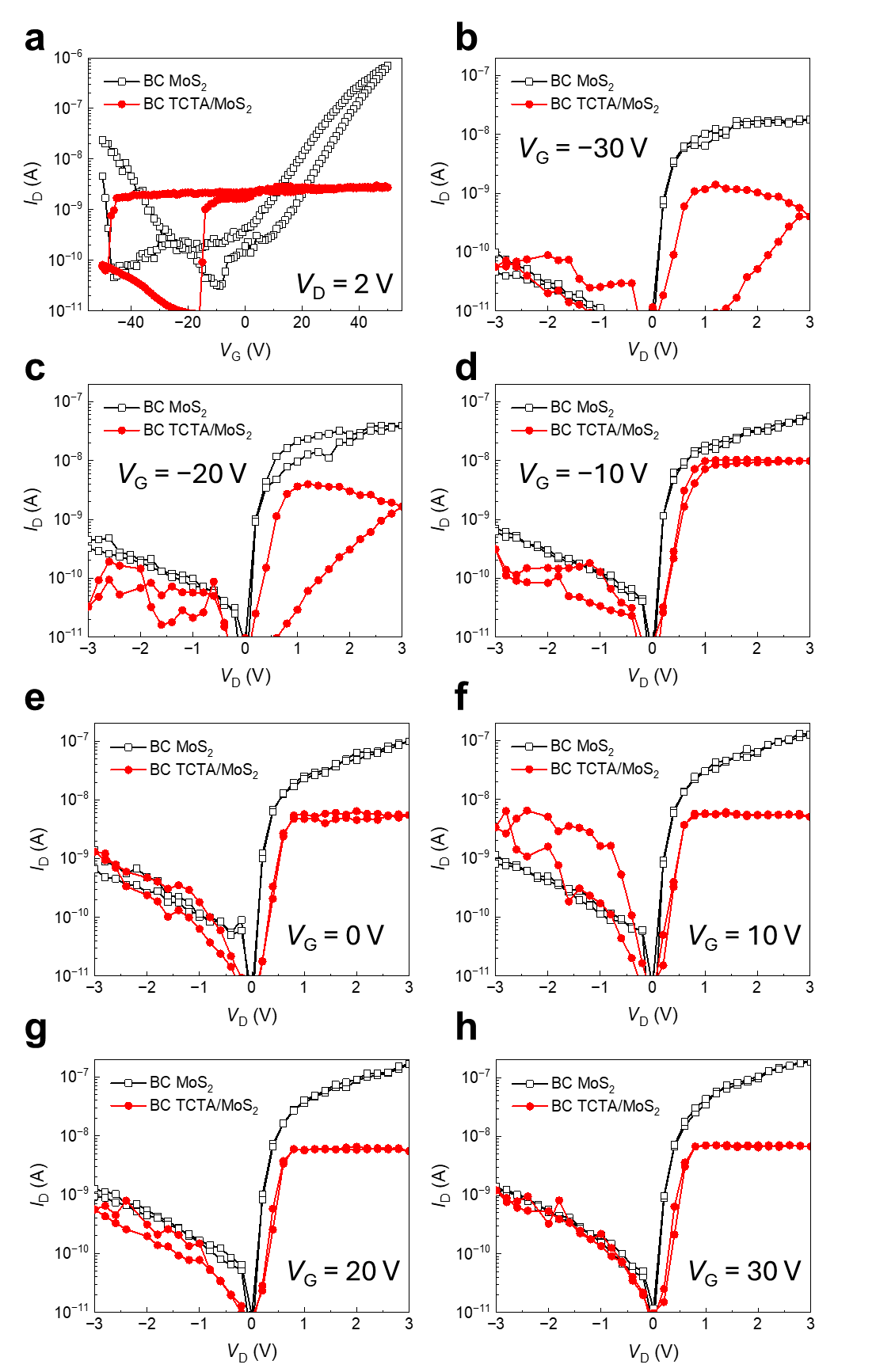


**Figure S7.** a) Transfer characteristic curves (*I*_D_–*V*_G_) of the BC MoS_2_-based FET at *V*_D_ = 2 V before (open black markers) and after (solid red markers) hybridization with TCTA. Output characteristic curves (*I*_D_–*V*_D_) of the BC MoS_2_-based FETs at various *V*_G_ as b) −30 V, c) −20 V, d) −10 V, e) 0 V, f) 10 V, g) 20 V, and h) 30 V, before (open black markers) and after (solid red markers) hybridization with TCTA.

Notably, memristive hysteresis characteristics were clearly observed for the BC TCTA/MoS_2_ FETs at negative *V*_G_ (–20 and –30 V) but not for the TC TCTA/MoS_2_ FET as shown in Figure S8.


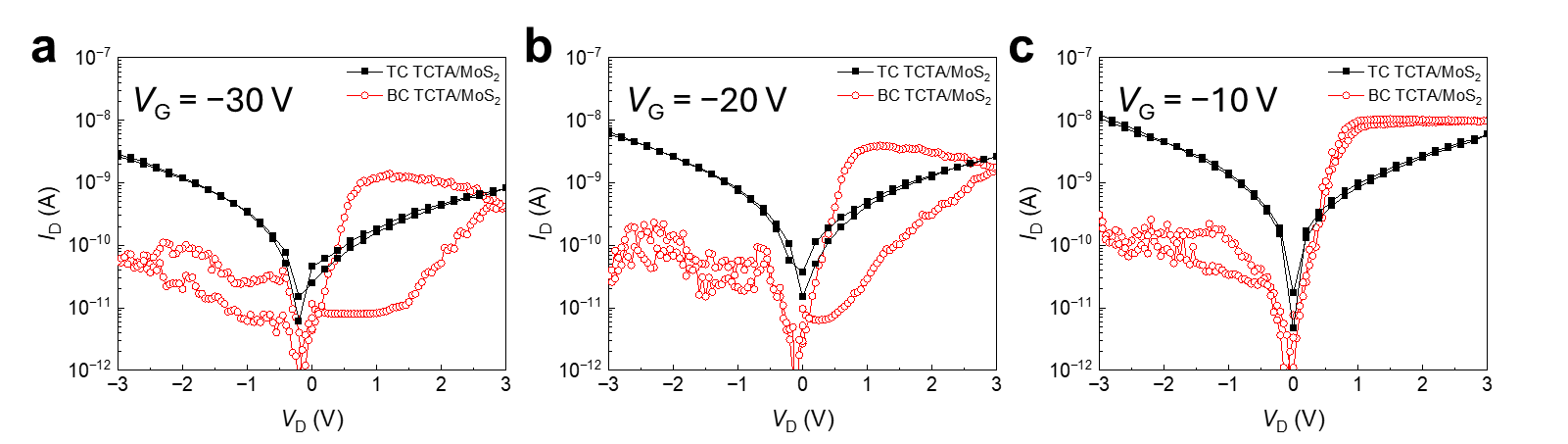


**Figure S8.** Comparative plots of the output characteristic curves of the TC and BC TCTA/MoS_2_ FETs at various *V*_G_ as a) −30 V, b) −20 V, and c) −10 V.

To further investigate the gate-dependent charge transport through the interface, the trap density (*N*_t_) was estimated using Figure S9 and the following Equation:^[S3]^

$N_{t}=\frac{\varepsilon_{0}\varepsilon_{\text{r}}}{eL^{2}}\left( \frac{l}{l+1} \right)\left( \frac{2l+1}{l+1} \right)^{\frac{l+1}{l}}V_{\text{TFL}}$,

where *V*_TFL_ represents the voltage at which the traps are fully filled. As *V*_G_ varied from −15 to −30 V, *V*_TFL_ and *N*_t_ increased from 0.36 V and 8.95 × 10^13^ cm^−3^ to 1.52 V and 4.14 × 10^14^ cm^−3^, respectively (Table S2). These results indicate that the negative gate bias enhanced both the current level and *N*_t_ in the active layer of TCTA/MoS_2_, suggesting a gate-dependent switching mechanism.


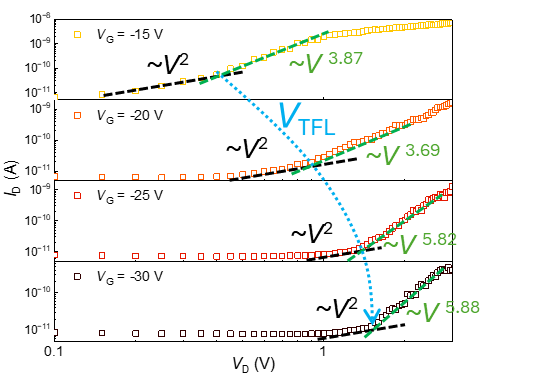


**Figure S9.** Output characteristics of BC TCTA/MoS_2_ at various *V*_G_. The cyan arrow provides a visual guide for the shift in *V*_TFL_.

**Table S2.** Estimated *V*_TFL_ and *N*_t_ values obtained from Figure S9.

| *V*_G_ (V) | −15 | −20 | −25 | −30 |
| --- | --- | --- | --- | --- |
| *V*_TFL_ (V) | 0.36 | 1.01 | 1.35 | 1.52 |
| *N*_t_ (cm^−3^) | 8.95 × 10^13^ | 2.47 × 10^14^ | 3.67 × 10^14^ | 4.14 × 10^14^ |

Figure S10 shows the analysis of charge transport mechanisms based on the Schottky conduction and SCLC models for the BC TCTA/MoS_2_ memtransistor. The corresponding coefficients of determination *R*^2^ are listed in Table S3. At *V*_G_ = 30 V, Schottky conduction dominated the charge transport mechanism. As *V*_G_ became increasingly negative, SCLC emerged as the dominant conduction mechanism, supporting the gate-dependent charge transport model.


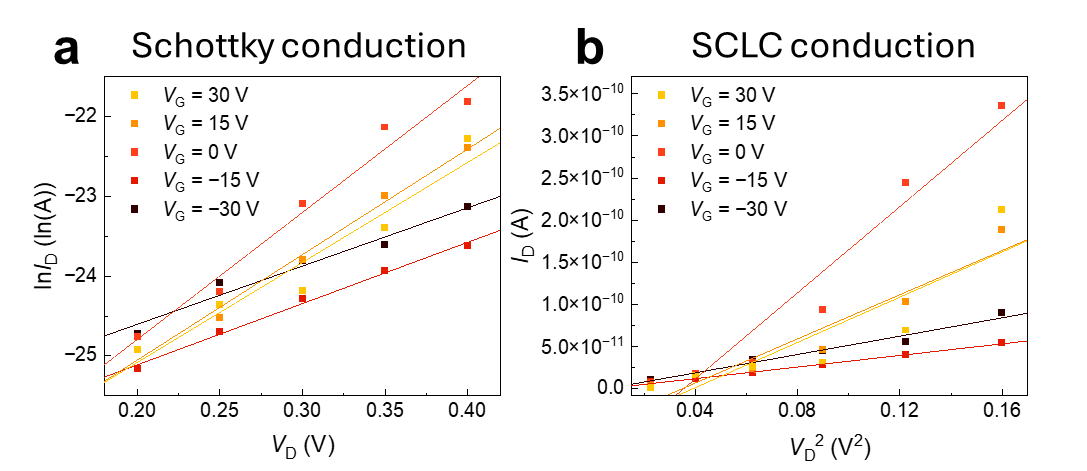


**Figure S10.** Comparative analysis of Schottky conduction and SCLC models. a) ln *I*_D_ vs. *V*_D_ for Schottky [*J* ~ exp(*V*)] conduction and b) *I*_D_ vs. *V*_D_^2^ for SCLC (*J* ~ *V*^2^) model plots obtained at various *V*_G_ (30, 15, 0, −15, and −30 V).

**Table S3.** Coefficients of determination. Estimated coefficients of determination *R*^2^ obtained from linear fitting of the plots in Figure S10.

| *V*_G_ (V) | 30 | 15 | 0 | −15 | −30 |
| --- | --- | --- | --- | --- | --- |
| *R*^2^_Schottky_ | 0.932 | 0.990 | 0.974 | 0.994 | 0.962 |
| *R*^2^_SCLC_ | 0.791 | 0.916 | 0.936 | 0.994 | 0.971 |

Figure S11a shows the *I*_D_–*V*_D_ output characteristic curves of the BC TCTA/MoS_2_ memtransistors in the current saturation region at various positive *V*_G_. Figure S11b shows the plot of *I*_sat_^1/2^ as a function of *V*_G_ with linear fitting.


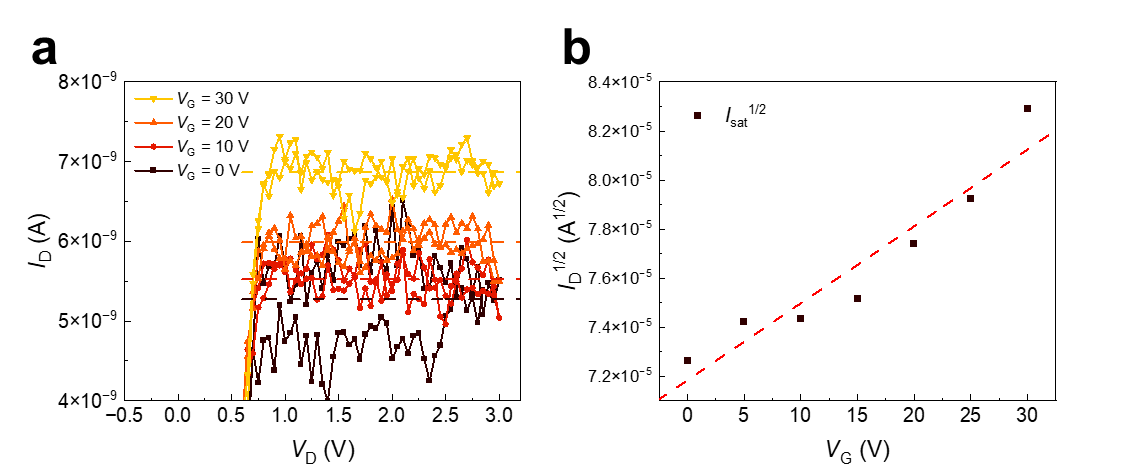


**Figure S11.** Saturation current regime in BC TCTA/MoS_2_ memtransistors. a) *I*_D_–*V*_D_ output characteristic curves in the current saturation region at various positive *V*_G_ (0, 10, 20, and 30 V). The values of saturated current (*I*_sat_) were obtained using constant fitting. b) Plot of *I*_sat_^1/2^ as a function of *V*_G_ with linear fitting.

**S-3. Synaptic function measurements.**

Figure S12 shows the measurement conditions and sequences for synaptic functions. For heterosynaptic (H-) LTP measurement (Figure S12a), a pre-synaptic pulse (*V*_pre_) was applied to the drain for write and a gate pulse (*V*_mod_) was applied for modulation prior to the readout pulse (*V*_read_). For H-STDP measurement (Figure S12b), paired pulses of *V*_pre_ and *V*_mod_ were synchronously applied between two *V*_read_ signals, and the time interval (Δ*t*) of paired pulses was systematically varied. These configurations enabled the observation of neuromorphic behavior as a function of modulated signal strength. For modulatory-induced homosynaptic (M-) measurements (Figure S12c and d), in contrast to the heterosynaptic measurements, no pre-synaptic pulse was applied; neuromodulation depended exclusively on the gate voltage. For M-LTP, drain current was measured following *V*_mod_. For M-STDP, Δ*t* between consecutive *V*_mod_ was modulated. These results confirm the neuromorphic characteristics induced by gate modulation.

The synaptic responses have been reproducible for the different batches of the BC TCTA/MoS_2_ memtransistors as shown in Figure S13, implying the reliability of neuromorphic electronics.


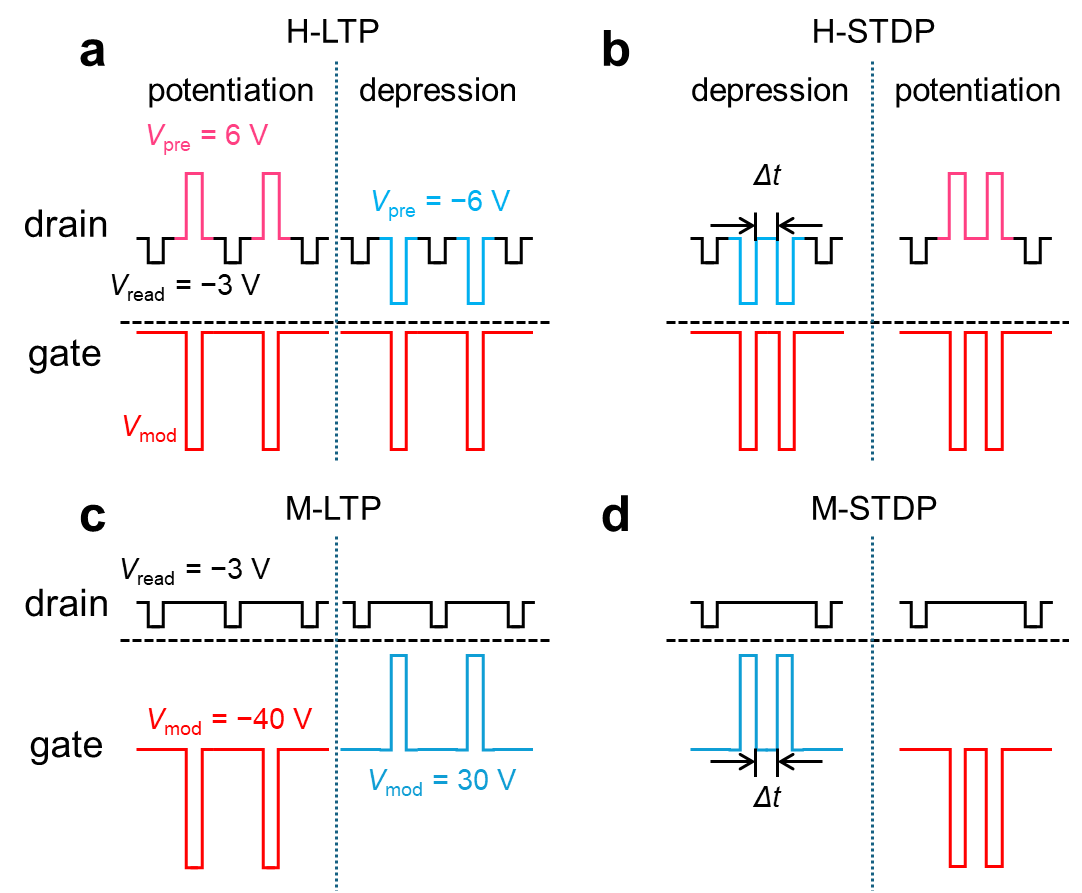


**Figure S12.** Measurement conditions and sequences for synaptic functions. Measurement sequences of heterosynaptic (H-) a) LTP and b) STDP. For potentiation, drain pulses (*V*_pre_) of 6 V were applied. *V*_pre_ = −6 V pulses were applied for depression. For both potentiation and depression, gate pulses (*V*_mod_) of −40, 0, and 30 V were applied. Measurement sequences of modulatory-induced homosynaptic (M-) c) LTP and d) STDP without *V*_pre_. *V*_mod_ was −40 V for potentiation and 30 V for depression. For all measurements, reading pulses (*V*_read_) of −3 V were applied to the drain. All pulse widths (*t*_width_) were fixed to 5 ms.


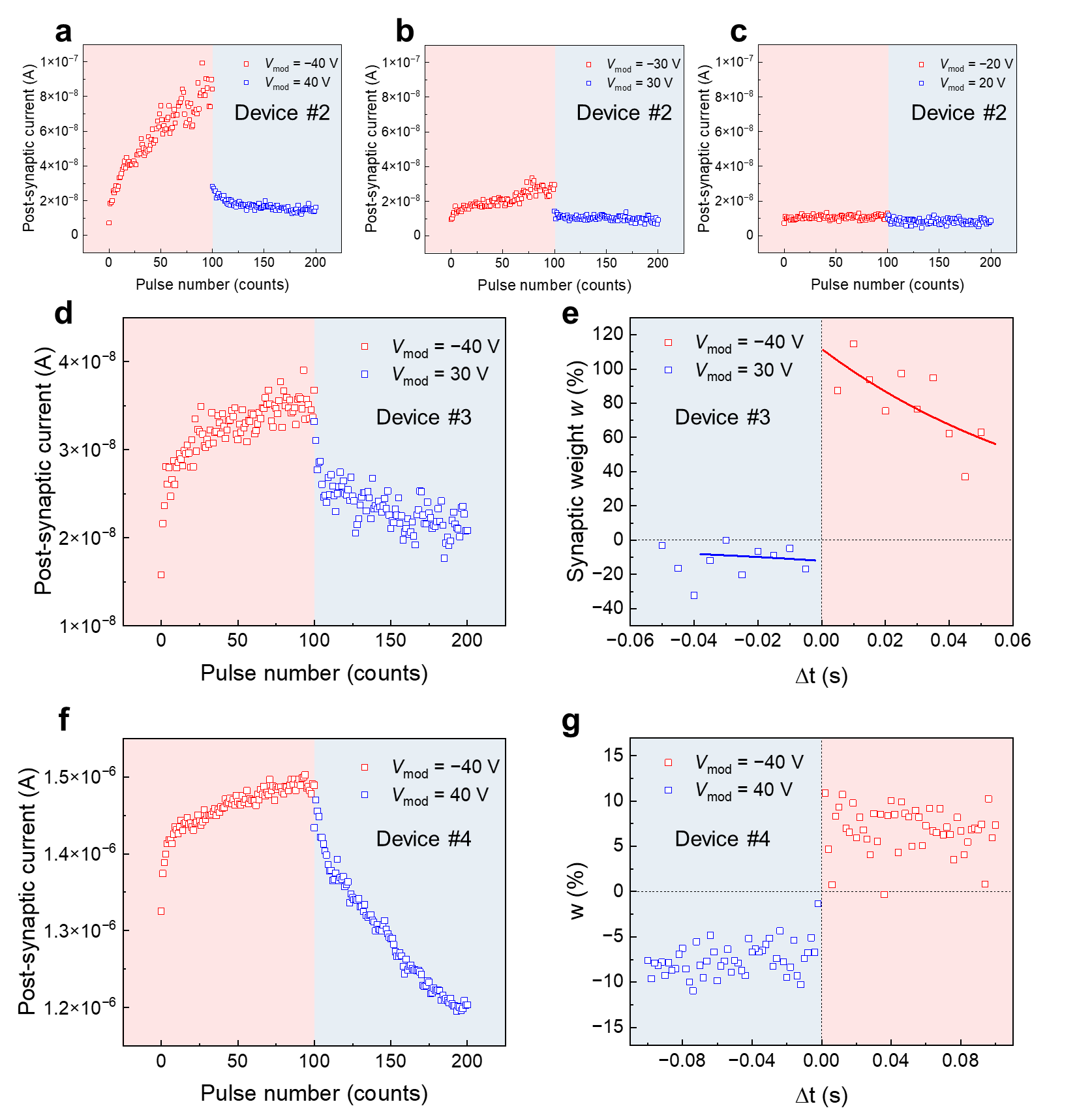


**Figure S13.** Synaptic responses in different batches of BC TCTA/MoS_2_ memtransistors. M-LTP obtained from a device #2 under *V*_mod_ = a) ±40 V, b) ±30 V, and c) ±20 V, with negative pulses for potentiation and positive one for depression. d) M-LTP and e) M-STDP obtained from a device #3 under *V*_mod_ = −40 V for potentiation and 30 V for depression. f) M-LTP and g) M-STDP obtained from a device #4 under *V*_mod_ = −40 V for potentiation and 40 V for depression. For all measurements, *V*_read_ = −3 V and *t*_width_ = 5 ms. The device numbering # (2, 3, and 4) indicates the different batches of BC TCTA/MoS_2_ memtransistors, which are also different from that in the main text (main device).

**S-4. Gate-induced memory characteristics.**

Figure S14a and b show the transfer characteristic curves of the BC MoS_2_ and TCTA/MoS_2_ FETs, respectively, with *V*_G_ swept from −30 to 30 V. First, *I*_D_ was measured without any modulation (black markers, denoted as “initial”). After 30 min of reset period (*t*_reset_), *V*_G_ = −60 V was applied for 15 s prior to the *I*_D_ measurement (red markers, denoted as “write”). Similarly, after *t*_reset_ = 30 min, *V*_G_ = 60 V was applied for 15 s and *I*_D_ was measured (blue markers, denoted as “erase”). These “write” and “erase” procedures were sequentially repeated 10 times. Lastly, after 30-min reset, *I*_D_ was measured without applying *V*_G_ (cyan markers, denoted as “final”). For BC MoS_2_ FETs (Figure S14a), *V*_th_ exhibited a slight shift. In contrast, the BC TCTA/MoS_2_ FETs (Figure S14b) displayed distinct hysteresis states depending on the pre-applied *V*_G_, corresponding to gate-induced memory characteristics. These results indicate that the conductance of BC TCTA/MoS_2_ FETs can be modulated by the polarity of *V*_G_ and highlight the potential of leveraging the gate-terminal as an additional input, corresponding to the polarity-dependent synaptic behavior of BC TCTA/MoS_2_ memtransistors.


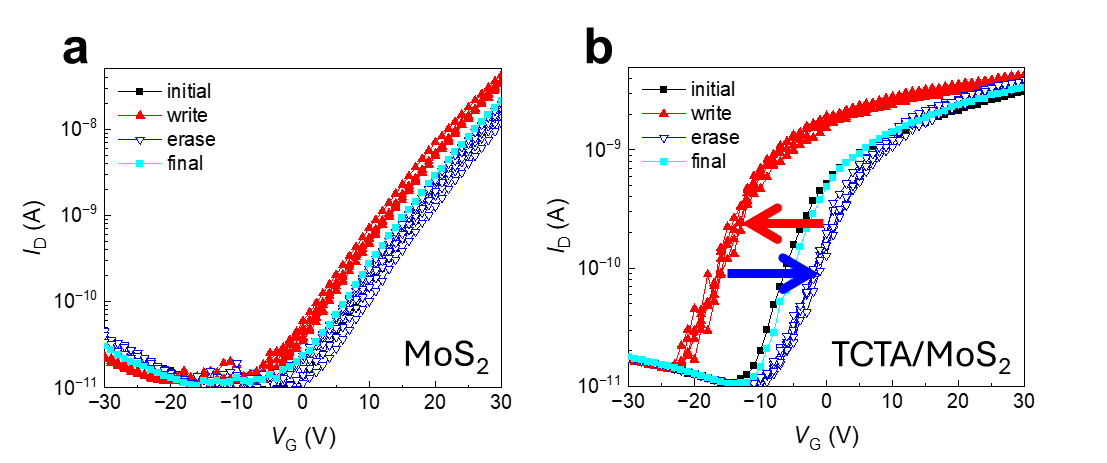


**Figure S14.** Gate-induced memory characteristics. Transfer characteristic curves of the BC a) MoS_2_ and b) TCTA/MoS_2_ FETs. All measurements were performed after a reset period of 30 min. The “initial” and “final” currents are the *I*_D_ measured without any modulation. “write” and “erase” were measured sequentially 10 times. Before measurements, *V*_G_ was applied for 15 s at −60 V for “write” (red markers) and +60 V for “erase” (blue markers) processes.

**References**

S1. M. Raoufi, S. Chandrabose, R. Wang, et al., “Influence of the Energy Level Alignment on Charge Transfer and Recombination at the Monolayer-MoS_2_/Organic Hybrid Interface,” *The Journal of Physical Chemistry C* (2023): 5866. https://doi.org/10.1021/acs.jpcc.2c08186

S2. J. Y. Kim, H. J. Park, S.-h. Lee, C. Seo, J. Kim, and J. Joo, “Distinctive Field-Effect Transistors and Ternary Inverters Using Cross-Type WSe_2_/MoS_2_ Heterojunctions Treated with Polymer Acid,” *ACS Applied Materials & Interfaces* (2020): 36530. https://doi.org/10.1021/acsami.0c09706

S3. Y. S. Shin, K. Lee, Y. R. Kim, et al., “Mobility Engineering in Vertical Field Effect Transistors Based on Van der Waals Heterostructures,” *Advanced Materials* (2018): 1704435. https://doi.org/10.1002/adma.201704435
